# Identification of a rare Gli1^+^ progenitor cell population contributing to liver regeneration during chronic injury

**DOI:** 10.1038/s41421-022-00474-3

**Published:** 2022-11-01

**Authors:** Jiayin Peng, Fei Li, Jia Wang, Chaoxiong Wang, Yiao Jiang, Biao Liu, Juan He, Kai Yuan, Chenyu Pan, Moubin Lin, Bin Zhou, Luonan Chen, Dong Gao, Yun Zhao

**Affiliations:** 1 State Key Laboratory of Molecular Biology, Shanghai Institute of Biochemistry and Cell Biology, Center for Excellence in Molecular Cell Science, Chinese Academy of Sciences, Shanghai, China; 2 University of Chinese Academy of Sciences, Beijing, China; 3 Department of General Surgery, Yangpu Hospital, Tongji University School of Medicine, Shanghai, China; 4 Institute for Stem Cell and Regeneration, Chinese Academy of Sciences, Beijing, China; 5 School of Life Science and Technology, ShanghaiTech University, Shanghai, China; 6 Key Laboratory of Systems Health Science of Zhejiang Province, School of Life Science, Hangzhou Institute for Advanced Study, University of Chinese Academy of Sciences, Hangzhou, Zhejiang China

**Keywords:** Regeneration, Transdifferentiation

## Abstract

In adults, hepatocytes are mainly replenished from the existing progenitor pools of hepatocytes and cholangiocytes during chronic liver injury. However, it is unclear whether other cell types in addition to classical hepatocytes and cholangiocytes contribute to hepatocyte regeneration after chronic liver injuries. Here, we identified a new biphenotypic cell population that contributes to hepatocyte regeneration during chronic liver injuries. We found that a cell population expressed Gli1 and EpCAM (EpCAM^+^Gli1^+^), which was further characterized with both epithelial and mesenchymal identities by single-cell RNA sequencing. Genetic lineage tracing using dual recombinases revealed that Gli1^+^ nonhepatocyte cell population could generate hepatocytes after chronic liver injury. EpCAM^+^Gli1^+^ cells exhibited a greater capacity for organoid formation with functional hepatocytes in vitro and liver regeneration upon transplantation in vivo. Collectively, these findings demonstrate that EpCAM^+^Gli1^+^ cells can serve as a new source of liver progenitor cells and contribute to liver repair and regeneration.

## Introduction

Functional tissue regeneration requires the coordinated action of distinct cell types. In addition, cell lineage conversion and cell plasticity have been identified in the context of regeneration in many severe injury models and in the progression of many diseases^1–4^. The liver is a complex organ composed of a heterogeneous mixture of epithelial and stromal lineages^5^. Different cell types in the liver may cooperate to carry out essential functions during homeostasis and after injury^6–8^. Therefore, identifying new cell types in the liver and their function in liver regeneration would provide tremendous insights into lineage plasticity in liver regeneration and therapeutic applications.

Hepatocytes and cholangiocytes are the only two epithelial cell lineages that enable the liver to exhibit a high regeneration capacity^9,10^. Lineage tracing of different types of liver cells demonstrated that hepatocytes could convert into mature cholangiocytes and form a functional and stable biliary system^11,12^. On the other hand, cholangiocytes could transdifferentiate into hepatocytes only when hepatocyte proliferation was completely suppressed, or in the context of long-term severe injury in mice^3,13^. In addition, Sox9^+^ hepatocytes, which are bipotent progenitors, produce both hepatocytes and ductal cells for liver repair and regeneration^14,15^. Moreover, hepatocytes exhibit extensive regenerative capacity in situ, rather than relying on rare specialized liver stem cells during liver repair and regeneration after injury^16–19^. In contrast, when hepatocyte and cholangiocyte proliferation is impaired in severe or chronic liver injury, facultative liver progenitor cells (LPCs) have been reported to contribute to regeneration. Previous studies suggested that Foxl1^+^ cells, which can produce hepatocytes after liver injury, could be recognized as the facultative progenitor cells^20,21^. Recently, single-cell RNA sequencing (scRNA-seq) was used to identify the EpCAM^+^TROP2^int^ cell population as a putative progenitor population in the adult human liver that was potentially involved in liver regeneration^8^. This evidence mainly focuses on the plasticity and contributions of epithelial cell lineages in liver regeneration. However, the role of nonepithelial populations is still largely unknown.

Previous studies have suggested that nonepithelial populations, such as mesenchymal cells and immune cells, reside near biliary ducts within periportal areas, and form a niche for LPCs during liver regeneration^9,20^. Mesenchymal cells are associated with migration to distant organs and the ability to maintain stemness, which could facilitate differentiation to multiple cell lineages during the initial stage of development and migration^20^. Mesenchymal cells are liver-resident mesenchymal stem cells (MSCs) due to their potential to differentiate into adipocytes or osteocytes^22,23^. Moreover, hepatic stellate cells (HSCs) have been identified as a source of LPCs, and could give rise to hepatocytes in the adult liver^24,25^. Mesenchymal cells within various tissues have been identified using a variety of markers including glioma-associated oncogene homolog 1 (Gli1), Grem1 and platelet-derived growth factor receptor α (PDGFRα)^26–28^. Gli1 identifies an MSC-like population in multiple tissues, and Gli1^+^ cells generate myofibroblasts during tissue fibrosis^27^. In the adult liver, Gli1^+^ cells are positioned around biliary ducts and undergo extensive expansion after injury^27^. Thus, Gli1 was selected as a mesenchymal cell marker to study the role of nonepithelial cells in liver regeneration.

To identify the role of nonepithelial populations in liver regeneration, we used genetic tracing, scRNA-seq, and organoid culturing to characterize adult liver cell populations marked by Gli1. We found that Gli1^+^ cells generated de novo hepatocytes in the liver after chronic injury. Genetic lineage tracing revealed that the Gli1^+^ nonhepatocyte population could generate hepatocytes after chronic liver injury using dual recombinases. Further examination revealed that Gli1^+^ cells in the liver are a heterogenous population that consists of PDGFRα^+^ stromal cells and EpCAM^+^PDGFRα^+^ biphenotypic cells. We demonstrate that EpCAM^+^Gli1^+^ cells can serve as a new source of liver progenitor cells and contribute to liver repair and regeneration.

## Results

### Gli1^+^ cells contribute to hepatocyte regeneration after chronic liver injury

In the liver, Gli1^+^ cells are located around biliary ducts and in the pericyte niche within periportal areas^27^. To characterize Gli1^+^ cells, we first examined Gli1-expressing cells and their location in the adult mouse liver. Consistent with a previous study, Gli1-expressing cells were present in the periportal areas of liver lobules in the adult mouse liver (Supplementary Fig. S1a). We then immunostained the liver sections of Gli1-LacZ mice for β-galactosidase (β-gal) and the biliary epithelial cell markers cytokeratin 19 (KRT19), epithelial cellular adhesion molecule (EpCAM) and osteopontin (OPN), the hepatocyte markers hepatocyte nuclear factor 4-alpha (HNF4α), Alb and glutamine synthetase (GS), the MSC marker PDGFRα, the activated HSC marker α-smooth muscle actin (α-SMA), the endothelial cell marker VE-cadherin (VE-CAD) and the macrophage marker F4/80 (Supplementary Fig. S1b, c). The results showed that Gli1^+^ cells in adult Gli1-lacZ mice livers were negative for the biliary epithelial cell markers KRT19 and OPN, the hepatocyte markers HNF4α, Alb, and GS, the endothelial cell marker VE-CAD and the macrophage marker F4/80 (Supplementary Fig. S1b). Strikingly, a subset of Gli1^+^ cells were positive for the MSC marker PDGFRα (78.01%) and the activated HSC marker α-SMA (11.19%) (Supplementary Fig. S1c). Interestingly, Gli1 also specifically labeled a minor subset of EpCAM^+^ cells (Supplementary Fig. S1b). Quantitatively, immunostaining showed that ~0.1% Gli1^+^ cells expressed EpCAM.

**Fig. 1:**
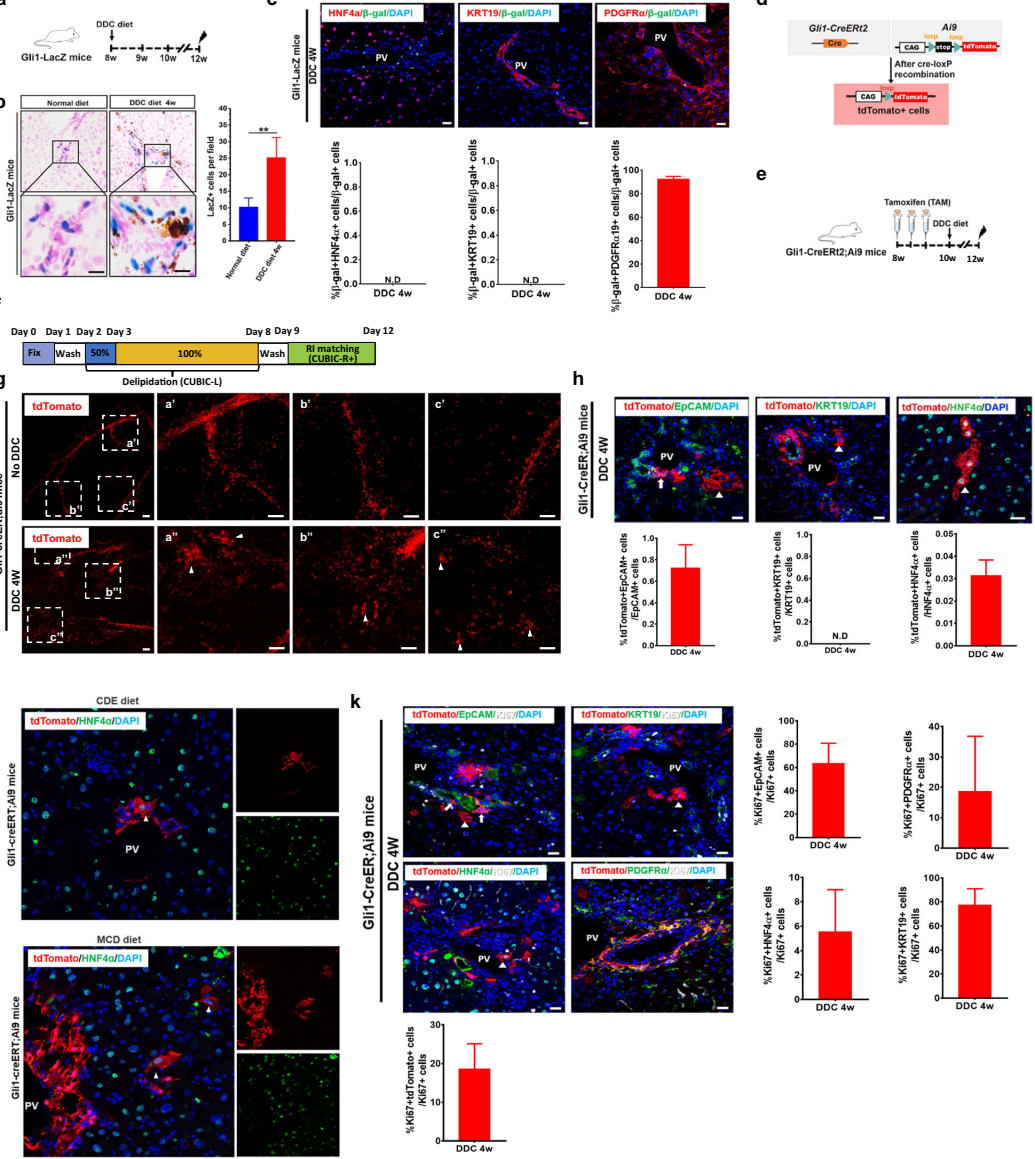
Gli1^+^ cells are induced and generate de novo hepatocytes after chronic liver injury. **a** Schematic illustration of the experimental design. **b** Representative histological images of X-gal-stained liver sections from Gli1-LacZ mice after 4 weeks of a normal diet or DDC diet (left panel). Quantification of the number of X-gal^+^ cells after injury (right panel). Data are shown as means ± SEM (*n* = 3). **P < 0.01. **c** Representative immunofluorescence staining of liver sections from mice that received a normal or DDC diet using antibodies against β-gal (green) and PDGFRα (red), HNF4α (red), or KRT19 (red). PV portal vein. Scale bars, 50 μm. Right panels: quantification of the percentage of β-gal^+^ cells expressing HNF4α, KRT19 and PDGFRα. Data are represented as means ± SEM (*n* = 5). N.D not detected. **d** Schematic representation of lineage tracing using Gli1-Cre^ERt2^;Ai9 reporter mice after DDC-induced liver injury. **e** Schematic illustration of the experimental design. **f** Schematic diagram of the protocol for clearing the liver from Gli1-Cre^ERt2^;Ai9 mice after 4 weeks of a normal diet or DDC diet with CUBIC. **g** Reconstructed 3D images of the livers of Gli1-Cre^ERt2^;Ai9 mice after 4 weeks of a normal diet or DDC diet. Images were obtained by light-sheet fluorescence microscopy (LSFM) (*z*-stack: 5 μm/slice). White arrowheads indicate hepatocyte morphology. Scale bars, 200 μm. **h** Immunostaining for tdTomato (red) and KRT19 (green), EpCAM (green) or HNF4α (green) in tissue sections from livers after Gli1-Cre^ERt2^;Ai9 mice received a DDC diet for 4 weeks. Scale bars, 50 μm. White arrowheads indicate hepatocyte morphology. White arrow indicates EpCAM^+^tdtomato^+^ cells. Right panels: quantification of the percentage of tdTomato^+^ cells expressing HNF4α, KRT19 and EpCAM. Data are shown as means ± SEM (*n* = 5). N.D not detected. *P < 0.05. **i** Immunostaining for tdTomato (red) and HNF4α (green) in tissue sections from livers after Gli1-Cre^ERt2^;Ai9 mice received an MCD diet for 4 weeks. White arrowhead indicates hepatocyte morphology. Scale bar, 50 μm. **j** Immunostaining for tdTomato (red) and HNF4α (green) in tissue sections from livers after Gli1-Cre^ERt2^;Ai9 mice received a CDE diet for 4 weeks. White arrowheads indicate hepatocyte morphology. Scale bar, 10 μm. **k** Immunostaining for tdTomato (red), Ki67 (white) and KRT19 (green), EpCAM (green), HNF4α (green) or PDGFRα (green) within representative liver sections at 4 weeks after DDC-induced injury. White arrowheads indicate hepatocyte morphology. White arrow indicates EpCAM^+^tdtomato^+^ cells. Scale bars, 50 μm. Right panels: quantification of the percentage of tdTomato^+^, KRT19^+^, EpCAM^+^, HNF4α^+^, or PDGFRα^+^ cells expressing Ki67. N.D, not detected. Data are shown as means ± SEM (*n* = 5).

To investigate the role of Gli1^+^ cells in liver regeneration, we used different models of liver injury in Gli1-LacZ mice. First, we investigated changes in Gli1^+^ cells in acute liver injury after partial hepatectomy (PH), which is a model of hepatocyte regeneration that is not associated with hepatocyte injury^29^. We collected liver samples at 24 and 48 h after surgery, and examined Gli1^+^ cells by X-gal staining. Notably, there was no significant increase in Gli1^+^ cells in the liver at 24 and 48 h after PH (Supplementary Fig. S1d). Next, we examined whether a single high dose of CCl_4_ (1 mL/kg, acute injury) led to the expansion of Gli1^+^ cells. The X-gal staining results also showed that Gli1^+^ cells did not increase 2 days after CCl_4_ injection (Supplementary Fig. S1e). However, a significant increase in Gli1^+^ cells was observed in the chronic liver injury mouse model which was injected with a low dose of CCl_4_ three times per week for 10 times (Supplementary Fig. S1f). Alternatively, we used the biliary injury model induced by a 3,5-diethoxycarboncyl-1,4-dihydrocollidine (DDC), methionine/choline-deficient (MCD) or choline-deficient, ethionine-supplemented (CDE) diet to study the cellular dynamics of Gli1^+^ cells during cholestatic injury (Fig. 1a, b; Supplementary Fig. S1g, h). Under these conditions, an increase in Gli1^+^ cells was apparent relative to that of mice fed a normal diet (Fig. 1b; Supplementary Fig. S1g, h). Consistent with the X-gal staining results, immunostaining of tissue sections for β-gal also showed that the DDC, MCD or CDE diet induced injury and stimulated significant expansion of Gli1^+^ cells (Supplementary Fig. S1i). Sectional staining of β-gal with cell lineage markers, such as the biliary epithelial cell marker KRT19, the hepatocyte marker HNF4α or the MSC marker PDGFRα, showed that 0%, 0%, or 97.24% of Gli1^+^ cells were KRT19^+^, HNF4α^+^, and PDGFRα^+^, respectively (Fig. 1c). These results indicated that Gli1^+^ cells were actively involved in chronic liver injuries.

To further investigate the role of Gli1-expressing cells in liver repair and regeneration, we performed genetic lineage tracing experiments using Gli1-Cre^ERt2^;Ai9 mice by administering tamoxifen (TAM) at 8 weeks (Fig. 1d). In a lineage tracing study, the level of background recombination caused by “leaky” expression of Cre was examined^30^. Here, we did not observe any “leaky” labeled cells after injury in the absence of TAM injection in Gli1-Cre^ERt2^;Ai9 mice (Supplementary Fig. S2a, b). The identity of lineage-marked tdTomato^+^ cells was characterized in Gli1-Cre^ERt2^;Ai9 adult mouse livers after 1 week, 4 weeks, 8 weeks or 24 weeks of TAM injection (Supplementary Fig. S3a). Gli1-marked tdTomato^+^ cells were also positioned around the biliary ducts of the liver, and no obvious difference was observed at different stages (Supplementary Fig. S3b–f). Injury was induced at least 14 days (a washout period) after TAM administration to eliminate the possibility of recombination after injury (Supplementary Fig. S3g). Consistent with the findings in Gli1-LacZ mice, we also observed a dramatic increase in tdTomato^+^ cells in the liver of Gli1-Cre^ERt2^;Ai9 mice after DDC-induced injury for 4 or 12 weeks (Supplementary Fig. S3h–j). Then, we applied a clear, unobstructed brain imaging cocktails and computational analysis (CUBIC) method, which is a tissue-clearing and three-dimensional (3D) imaging technique, to perform an experimental study (Fig. 1e, f). Whole-liver 3D images were collected by light-sheet microscopy and used for whole-liver comparisons between the livers of normal and DDC-induced Gli1-Cre^ERt2^;Ai9 mice. In the injury group, tdTomato^+^ cells showed typical hepatocyte morphology, and the cells were clustered or singly distributed (Fig. 1g). To examine the detailed cell types labeled by Gli1 in the liver, we sectioned the liver after DDC-induced injury and immunostained for tdTomato and cell lineage markers. We found that a subset of tdTomato^+^ cells in the liver colocalized with HNF4α or EpCAM. Quantitative analysis showed that 0.032%, 0% and 0.72% of tdTomato^+^ cells expressed HNF4α, KRT19 or EpCAM, respectively (Fig. 1h; Supplementary Fig. S3k). To accurately detect these events in the liver, 50 μm cryosections were obtained and stained according to standard immunofluorescence staining procedures. Finally, immunostaining images were acquired by a confocal microscope (Supplementary Fig. S3l). We found > 85% of tdTomato^+^ hepatocyte adjacent to the portal vein (PV) (Supplementary Fig. S3m). In addition, we also used the CDE or MCD diet to induce a cholestatic injury model (Fig. 1i, j). Consistent with the findings in DDC-induced injury, we found that a subset of tdTomato^+^ cells in the liver colocalized with HNF4α (Fig. 1i, j). However, no tracing events were detected with the same genotype in livers with a low dose of CCl_4_-induced chronic injury. We hypothesize that CCl_4_ induces central vein damage, but DDC, CDE or MCD causes the emergence of LPCs^21^. Next, to examine whether tdTomato^+^ cells could proliferate under these chronic liver injury conditions, we performed immunostaining for Ki67/tdTomato and triple-staining for various markers (including the cholangiocyte markers KRT19 and EpCAM, the hepatocyte marker HNF4α and the MSC marker PDGFRα). Quantitatively, immunostaining showed that 18.2%, 63.5%, 77.3%, 5.5%, and 18.6% of Ki67^+^ cells expressed tdTomato, EpCAM, KRT19, HNF4α and PDGFRα, respectively. A higher proliferation rate of Ki67^+^ cells was observed in EpCAM^+^, KRT19^+^, and PDGFRα^+^ cells treated with DDC (Fig. 1k). These data demonstrated that Gli1-derived cells contributed to liver regeneration after chronic injury.

### Hepatocytes derived from Gli1^+^ nonhepatocytes after chronic injury

**Fig. 2:**
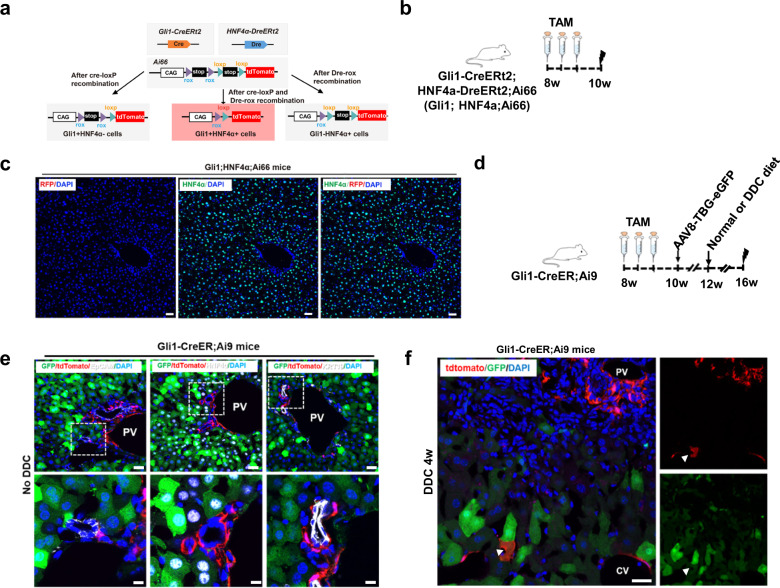
Hepatocytes derived from Gli1^+^ nonhepatocytes upon chronic injury. **a** Schematic diagram showing the strategy for labeling Gli1^+^HNF4α^+^ cells by a dual recombinase-mediated intersectional genetic approach. **b** Schematic illustration of the experimental design. **c** Immunostaining for tdTomato (red) and HNF4α (green) in representative liver sections after DDC-induced injury. Scale bars, 50 μm. **d** Schematic illustration of the experimental design. **e** Immunostaining for GFP (green), tdTomato (red) and EpCAM (white), HNF4α (white) or KRT19 (white) in liver sections from Gli1-Cre^ERt2^;Ai9 mice. PV portal vein. Scale bars, 50 μm. **f** Immunostaining for GFP (green) and tdTomato (red) in liver sections from Gli1-Cre^ERt2^;Ai9 mice after DDC-induced injury and AAV8-TBG-GFP viral particle injection. White arrowheads indicate hepatocyte morphology. Scale bar, 50 μm.

To identify Gli1^+^ cells that contribute to hepatocyte regeneration, we crossed HNF4α-Dre^ERt2^ and Gli1-Cre^ERt2^ mice with dual recombinase-mediated tdTomato reporter strain (Ai66) to generate the Gli1-Cre^ERt2^;HNF4α-Dre^ERt2^;Ai66 mouse line (Fig. 2a). In this reporter line, tdTomato activation required both Dre-rox and Cre-loxP recombination, and only Gli1^+^HNF4α^+^ cells, could be genetically labeled (Fig. 2a, b)^14^. We did not detect any tdTomato^+^ hepatocytes in the Gli1-Cre^ERt2^;HNF4α-Dre^ERt2^;Ai66 mice with or without injury, demonstrating that no Gli1^+^HNF4α^+^ hepatocytes were labeled (Fig. 2c). To avoid potential bias introduced by TAM injection, we next labeled hepatocytes with AAV8-TBG-GFP, which is known to strongly label hepatocytes. We inserted GFP into an AAV8 vector downstream of a *thyroxine-binding globulin* (*TBG*) promoter to overexpress GFP in hepatocytes. After TAM treatment and a 2-week washout period, Gli1-Cre^ERt2^;Ai9 mice were injected with 100 μL of AAV8-TBG-GFP viral particles (2 × 10^11^) through the tail vein with BD ultra-fine insulin syringes (Fig. 2d). Immunostaining of liver sections for tdTomato, GFP and HNF4α, EpCAM or KRT19 showed that GFP labeled 99% of hepatocytes; however, no tdTomato^+^ hepatocytes were observed in these experiments (Fig. 2e). Next, we performed genetic lineage tracing experiments using Gli1-Cre^ERt2^;Ai9 mice by administering TAM and AAV8-TBG-GFP viral particles upon chronic injury. We observed the presence of tdTomato^+^GFP^+^ cells in the livers of Gli1-Cre^ERt2^;Ai9 mice after DDC-induced injury for 4 weeks (Fig. 2f). Taken together, these data demonstrate that Gli1^+^ nonhepatocytes contribute to hepatocyte regeneration upon chronic injury.

### Gli1 labels a minor subset of EpCAM^+^ cells in the liver

**Fig. 3:**
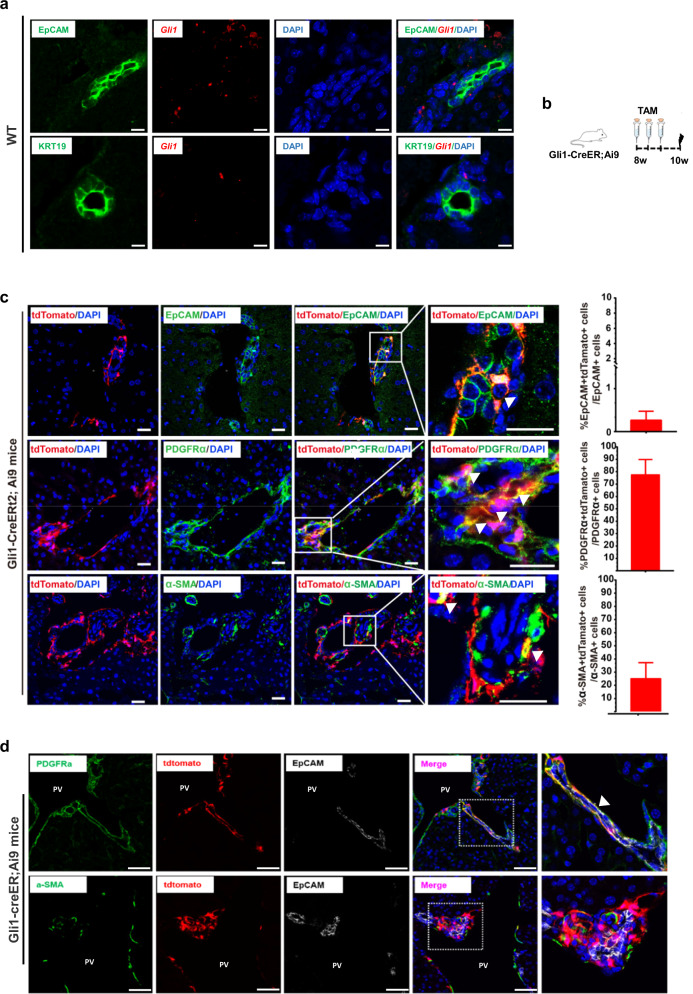
Gli1 identifies a minor subset of EpCAM^+^ cells in the liver. **a** RNAscope analysis of hepatic expression of Gli1 (red) and EpCAM (green) or CK19 (green) on 8-week-old WT mice. Scale bars, 200 μm. **b** Experimental design for lineage tracing of Gli1^+^ cells using Gli1-Cre^ERt2^;Ai9 mice. **c** Immunostaining for tdTomato (red) and EpCAM (green), PDGFRα (green) or α-SMA (green) on liver sections (left panels). White arrowheads indicate the co-stained cells. Scale bars, 50 μm. Quantification of the percentage of tdTomato^+^ cells expressing PDGFRα, EpCAM, and α-SMA (right panels). Data are presented as means ± SEM (*n* = 3). **d** Immunostaining for tdTomato (red), EpCAM (white), and PDGFRα (green) or α-SMA (green) in liver sections. PV portal vein. White arrowhead indicates the co-stained cell. Scale bars, 50 μm.

To further characterize Gli1^+^ cells, we performed *Gli1* FISH and EpCAM or KRT19 co-staining on 8-week-old wild-type (WT) mice. We observed that Gli1-expressing cells were located near the PV in the adult liver and Gli1 colocalized with EpCAM but not KRT19 (Fig. 3a; Supplementary Fig. S4a). Next, we also examined the detailed cell type of Gli1-expressing cells and their location in livers (Fig. 3b). Immunostaining of liver sections for tdTomato, PDGFRα and α-SMA showed that 77.8% and 25.18% of tdTomato^+^ cells were PDGFRα^+^ and α-SMA^+^ cells, respectively (Fig. 3c). Notably, Gli1 also specifically labeled a very small subset of EpCAM^+^ (0.26%) cells (Fig. 3c), which was consistent with the findings in Gli1-LacZ mice. We also stained for other cell lineage markers, including KRT19, Sox9, F4/80, Alb, HNF4α, and VE-CAD, with tdTomato in liver sections, and we did not find any tdTomato^+^ cells that were positive for these cell lineage markers (Supplementary Fig. S4b, c). Flow cytometric analysis of cells isolated from livers also showed that ~0.037% of tdTomato^+^ cells in the livers were EpCAM^+^ (Supplementary Fig. S4d). Moreover, we performed Gli1/EpCAM/PDGFRα or Gli1/EpCAM/α-SMA triple-staining to characterize the lineage of EpCAM^+^Gli1^+^ cells in Gli1-Cre^ERt2^;Ai9 mice. The results showed that 80.25% of EpCAM^+^Gli1^+^ cells were PDGFRα^+^ but not α-SMA^+^ (Fig. 3d). Taken together, these data demonstrate that EpCAM^+^Gli1^+^ cells are a subset of PDGFRα^+^ mesenchymal cells. It should be noted that EpCAM^+^ cells are often identified as potential liver stem cell populations^5,31^, suggesting that EpCAM^+^ cells may have different properties and functional heterogeneity in liver injury.

### EpCAM^+^Gli1^+^ cells exhibit a biphenotypic state and coexpress epithelial and mesenchymal markers

**Fig. 4:**
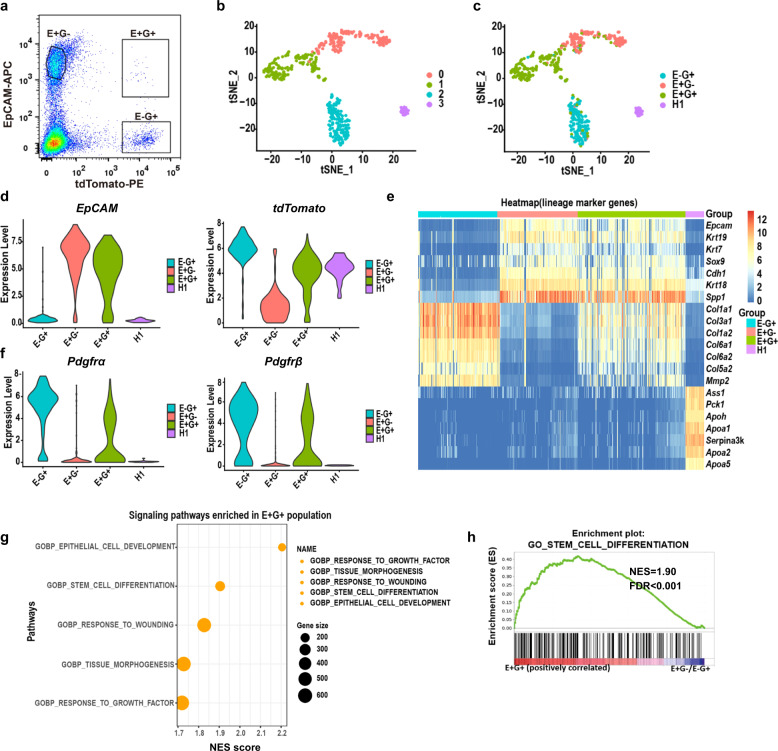
Single-cell transcriptome analysis identifies cell lineages expressing EpCAM and Gli1. **a** Representative profiles of FACS-sorted EpCAM^+^Gli1^−^, EpCAM^−^Gli1^+^ and EpCAM^+^Gli1^+^ cell populations from Gli1-Cre^ERt2^;Ai9 mice for scRNA-seq. **b**, **c** t-stochastic neighbor embedding (tSNE) plot of 527 individual cells isolated in **a** (dots). **d** Violin plots showing expression of EpCAM and tdTomato as determined by scRNA-seq. E+G− EpCAM^+^Gli1^−^, E−G+ EpCAM^−^Gli1^+^, E+G+ EpCAM^+^Gli1^+^, H1 hepatocyte. **e** Heatmap of scRNA-seq data showing enriched genes in the 4 different clusters. **f** Violin plots showing expression of selected specific lineage-associated genes as determined by scRNA-seq. **g** GO annotation of EpCAM^+^Gli1^+^ cells. **h** Signaling pathways that were significantly enriched in EpCAM^+^Gli1^+^ cells were identified using BioCarta gene sets for GSEA. *P* < 0.05. E+G− EpCAM^+^Gli1^−^, E−G+ EpCAM^−^Gli1^+^, E+G+ EpCAM^+^Gli1^+^, H1 hepatocyte.

EpCAM^+^ cells are comprised of an ASGR1^+^ hepatocyte population, a cholangiocyte population, and a putative progenitor cell population in the adult liver^8^. To determine the identity of EpCAM^+^Gli1^+^ cells, we performed scRNA-seq of individual cells isolated from Gli1-Cre^ERt2^;Ai9 mouse livers. EpCAM^−^Gli1^+^ cells, EpCAM^+^Gli1^−^ cells and EpCAM^+^Gli1^+^ cells were isolated from Gli1-Cre^ERt2^;Ai9 mouse livers (Fig. 4a). Hepatocytes from HNF4α-Dre^ERt2^;R26-RSR-tdTomato mice were selected as the negative control. We analyzed the scRNA-seq profiles of 527 cells (EpCAM^−^Gli1^+^ cells, *n* = 144; EpCAM^+^Gli1^−^ cells, *n* = 148; EpCAM^+^Gli1^+^ cells, *n* = 202; hepatocytes, *n* = 33) using Smart-seq2 methods. Doublets were identified and filtered by DoubletDecon and DoubletFinder (Supplementary Fig. S5a). Unsupervised clustering and principal component analysis (PCA) resulted in four clusters (clusters 1–4) (Fig. 4b; Supplementary Fig. S5b). These four clusters were clearly identified as EpCAM^+^Gli1^−^, EpCAM^−^Gli1^+^, EpCAM^+^Gli1^+^ and hepatocytes, indicating the different biological features of these cells (Fig. 4c). We noticed that EpCAM was mainly expressed by EpCAM^+^Gli1^−^ cells and EpCAM^+^Gli1^+^ cells, whereas tdTomato was mainly expressed by EpCAM^−^Gli1^+^ cells and EpCAM^+^Gli1^+^ cells (Fig. 4d). In addition, hepatocytes also expressed tdTomato, which was used as a control (Fig. 4d). These results were consistent with the fluorescence-activated cell sorting (FACS) results and provided evidence for the good quality of our sequencing data. Then, we examined the marker genes enriched for each of the cell populations by cluster analysis and representative differentially expressed genes (DEGs) per cell population by heatmap (Fig. 4e). Hepatocytes specifically expressed hepatocyte marker genes, such as *Alb* and *Cyp7a1* (Supplementary Fig. S5c). EpCAM^−^Gli1^+^ cells highly expressed extracellular matrix (ECM) genes (*Col1a1*, *Col1a2*, *Col3a1*, and *Dcn*) and genes related to mesenchymal cell markers (*Pdgfrα* and *Pdgfrβ*), which are hallmarks of fibroblast-like cells and mesenchymal cells (Fig. 4e, f). Interestingly, we found that the expression of the marker genes in EpCAM^+^Gli1^+^ cells was between those in EpCAM^+^Gli1^−^ and EpCAM^−^Gli1^+^ cells. EpCAM^+^Gli1^+^ cells expressed both epithelial and mesenchymal marker genes, suggesting that the EpCAM^+^Gli1^+^ cells might perform the functions of these two kinds of cells (Fig. 4e, f; Supplementary Fig. S5d–f). To investigate the biological functions of EpCAM^+^Gli1^+^ cells, we performed Gene Ontology (GO) analysis of DEGs in EpCAM^+^Gli1^+^ cells. The results revealed several significantly enriched biological processes associated with tissue morphogenesis, the response to wounding and stem cell differentiation (Fig. 4g). By using gene set enrichment analysis (GSEA) to characterize the function of the EpCAM^+^Gli1^+^ cell subpopulation, we also found that tissue morphogenesis, the response to wounding and stem cell differentiation pathways were enriched in EpCAM^+^Gli1^+^ cells compared with the other three types of cells (Fig. 4h; Supplementary Fig. S5g). The coexpression of epithelial markers (*EpCAM* and *Krt7*) and mesenchymal markers (*Pdgfrα* and *Pdgfrβ*) supported the progenitor characteristics of the EpCAM^+^Gli1^+^ cell population, indicating that EpCAM^+^Gli1^+^ cells may be the source of Gli1^+^ hepatocytes after liver injury.

### Gli1^+^ biphenotypic cells generate de novo hepatocytes after chronic liver injury

**Fig. 5:**
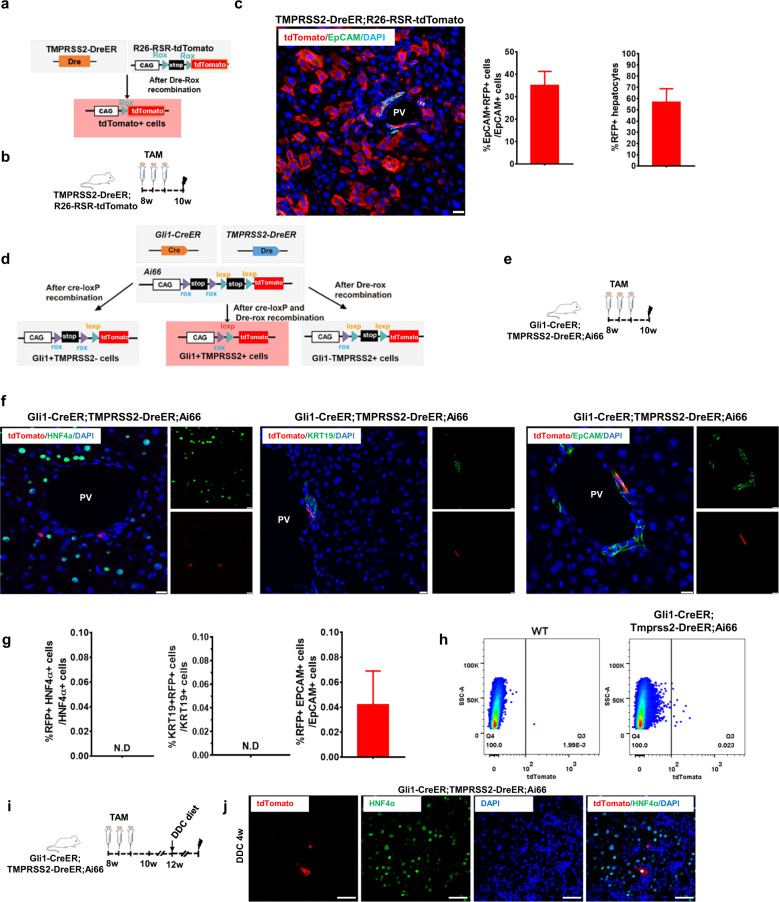
Gli1^+^ biphenotypic cells contribute to the formation of new hepatocytes for liver repair upon chronic injury. **a** Schematic diagram showing the lineage tracing strategy by Dre-rox recombination using Tmprss2-Dre^ERt2^;R26-RSR-tdTomato mice. **b** Schematic illustration of the experimental design. **c** Immunostaining for tdTomato (red) and EpCAM (green) in liver sections from Tmprss2-Dre^ER^;R26-tdTomato mice and the quantification of the percentage of tdTomato^+^ cells among EpCAM^+^ cells. PV portal vein. Scale bar, 50 μm. Data are shown as means ± SEM (*n* = 5). **d** Schematic diagram showing the lineage tracing strategy by Dre-rox and Cre-loxP recombination using Gli1-Cre^ERt2^;Tmprss2-Dre^ERt2^;Ai66 mice. **e** Schematic illustration of the experimental design. **f** Immunostaining for tdTomato (red) and HNF4α (green), KRT19 (green) or EpCAM (green) in liver sections from Gli1-Cre^ERt2^;Tmprss2-Dre^ERt2^;Ai66 mice. Scale bar, 50 μm. **g** Quantification of the percentage of tdTomato^+^ cells among EpCAM^+^, KRT19^+^ or HNF4α^+^ cells. Data are shown as means ± SEM (*n* = 5). **h** Flow cytometric analysis of the percentage of tdTomato^+^ cells. **i** Schematic illustration of the experimental design. **j** Immunostaining for tdTomato (red) and HNF4α (green) in liver sections from Gli1-Cre^ERt2^;Tmprss2-Dre^ERt2^;Ai66 mice after DDC-induced injury. Scale bars, 50 μm.

To achieve specific labeling of EpCAM^+^Gli1^+^ cells, we generated two distinct mouse lines: EpCAM-Cre^ERt2^ and Gli1-Dre^ERt2^ mice. The EpCAM-Cre^ERt2^ mouse was crossed with the Ai9 reporter mouse to generate the EpCAM-Cre^ERt2^;Ai9 mouse (Supplementary Fig. S6a). TAM induction led to Cre-loxP recombination, which resulted in permanent labeling of EpCAM^+^ cells and all their descendants. To examine tdTomato expression in adult tissues, we treated 8-week-old EpCAM-Cre^ERt2^;Ai9 mice with TAM and collected tissue samples 7 days later for analysis (Supplementary Fig. S6b). The majority of EpCAM^+^ cells were tdTomato^+^ in intestinal tissue, and the percentage of tdTomato^+^EpCAM^+^ cells was 95.26% (Supplementary Fig. S6c). In contrast, immunostaining showed that 21.96% of EpCAM^+^ cells expressed tdTomato in the liver (Supplementary Fig. S6d). The dual recombinase-mediated genetic approach has significantly enhanced the precision of in vivo lineage tracing, as well as gene manipulation. However, this approach is limited by the labeling efficiency of Cre and Dre recombinases. Since the labeling efficiency of EpCAM-Cre^ERt2^ is not high in the liver, we did not attempt to construct EpCAM-Dre^ERt2^ mice. We needed to find a gene that had the same expression pattern as EpCAM and could replace EpCAM for use in the dual recombinase system. Transmembrane protease serine 2 (Tmprss2) is a 70-kDa serine protease family member that is associated with physiological and pathological processes such as digestion, tissue remodeling, blood coagulation, fertility, inflammatory responses, tumor cell invasion and apoptosis^32^. *Tmprss2* mRNA is expressed in many tissues, including the prostate, breast, liver and lung. Tmprss2 protein and mRNA are mostly expressed in epithelial cells^32^. In the liver, lineage tracing studies using Tmprss2-Cre^ERt2^;R26-YFP mice showed that the Tmprss2 protein was localized to the two main epithelial cell types: hepatocytes and ductal cells (Supplementary Fig. S6e). Recent research indicated that Tmprss2 was specifically coexpressed in TROP2^**+**^ liver progenitors in human liver tissue using scRNA-seq and FISH^33^. The results indicated that Tmprss2 was specifically present in liver progenitors with a cholangiocyte fate bias. Based on these data, the *Tmprss2* gene can be exploited to solve the problem of labeling Gli1^+^ biphenotypic cells. For lineage tracing of Tmprss2^+^ cells, we generated a new mouse line (Tmprss2-Dre^ERt2^) by targeting cDNA encoding the Dre protein fused to the *Tmprss2* gene locus to replace the endogenous translational start codon ATG, followed by woodchuck posttranscriptional regulatory element (WPRE) and a polyA sequence. The Tmprss2-Dre^ERt2^ mouse was crossed with the R26-RSR-tdTomato reporter mouse to generate the Tmprss2-Dre^ERt2^;R26-RSR-tdTomato mouse (Fig. 5a). TAM induction led to Dre-loxP recombination, which resulted in permanent labeling of Tmprss2^+^ cells and all their descendants (Fig. 5b). Immunostaining of Tmprss2-Dre^ERt2^;R26-RSR-tdTomato liver sections for EpCAM showed that 63.08% of EpCAM^+^ cells were tdTomato^+^ (Fig. 5c). The tdTomato staining was specific because we did not detect it in tissues collected from oil-treated Tmprss2-Dre^ER^;R26-RSR-tdTomato mice (Supplementary Fig. S6f). To achieve specific labeling of Gli1^+^Tmprss2^+^ cells, we crossed Tmprss2-Dre^ERt2^ and Gli1-Cre^ERt2^ mice with a dual recombinase-mediated tdTomato reporter (Ai66) to generate the Gli1-Cre^ERt2^;Tmprss2-Dre^ERt2^;Ai66 mouse line (Fig. 5d). Two weeks after TAM induction, livers were collected from Gli1-Cre^ERt2^;Tmprss2-Dre^ERt2^;Ai66 mice for analysis (Fig. 5e). Immunostaining of liver sections for HNF4α, KRT19 or EpCAM showed that 0.042% of EpCAM^+^ cells were tdTomato^+^, while HNF4α hepatocytes and KRT19^+^ cells were tdTomato^−^ (Fig. 5f, g), demonstrating selective labeling of EpCAM+ cells. Flow cytometric analysis showed that ~0.023% of EpCAM^+^ cells were labeled with tdTomato (Fig. 5h). We also examined the littermate controls (Gli1-Cre^ERt2^;Ai66 or Tmprss2-Dre^ERt2^;Ai66) and performed the same TAM induction strategy and immunostaining for subsequent analysis. In both groups, no tdTomato^+^ cells were detected, demonstrating that tdTomato expression required both Cre and Dre recombination. Taken together, these data demonstrated the successful generation of a genetic tool for targeting Gli1^+^ Tmprss2^+^ cells in the liver. To examine the dynamics of Gli1^+^Tmprss2^+^ cells after liver injury, we fed Gli1-Cre^ERt2^;Tmprss2-Dre^ERt2^;Ai66 mice with DDC diet to induce liver injury after TAM induction. After 4 weeks, liver tissues were collected for analysis (Fig. 5i). We observed specific labeling of tdTomato^+^HNF4α^+^ hepatocytes in the injured liver (Fig. 5j). In addition, we also performed genetic lineage tracing experiments using EpCAM-Cre^ERt2^;Ai9 or PDGFRα-Cre^ERt2^;Ai9 mice by administering TAM. Following 14 days, DDC was adopted to induce liver injury. Consistent with the findings in Gli1-Cre^ERt2^;Ai9 mice, we also observed a subset of tdTomato^+^ cells in the liver that were co-stained by HNF4α (Supplementary Fig. S6i). Quantitative analysis showed that ~0.059% or ~0.075% of tdTomato^+^ cells expressed HNF4α, respectively (Supplementary Fig. S6h, j). Taken together, these data demonstrated that Gli1^+^Tmprss2^+^ contributed to de novo hepatocyte production after DDC-induced injury.

### EpCAM^+^Gli1^+^ cells form liver organoids and can differentiate into functional hepatocytes in vitro

**Fig. 6:**
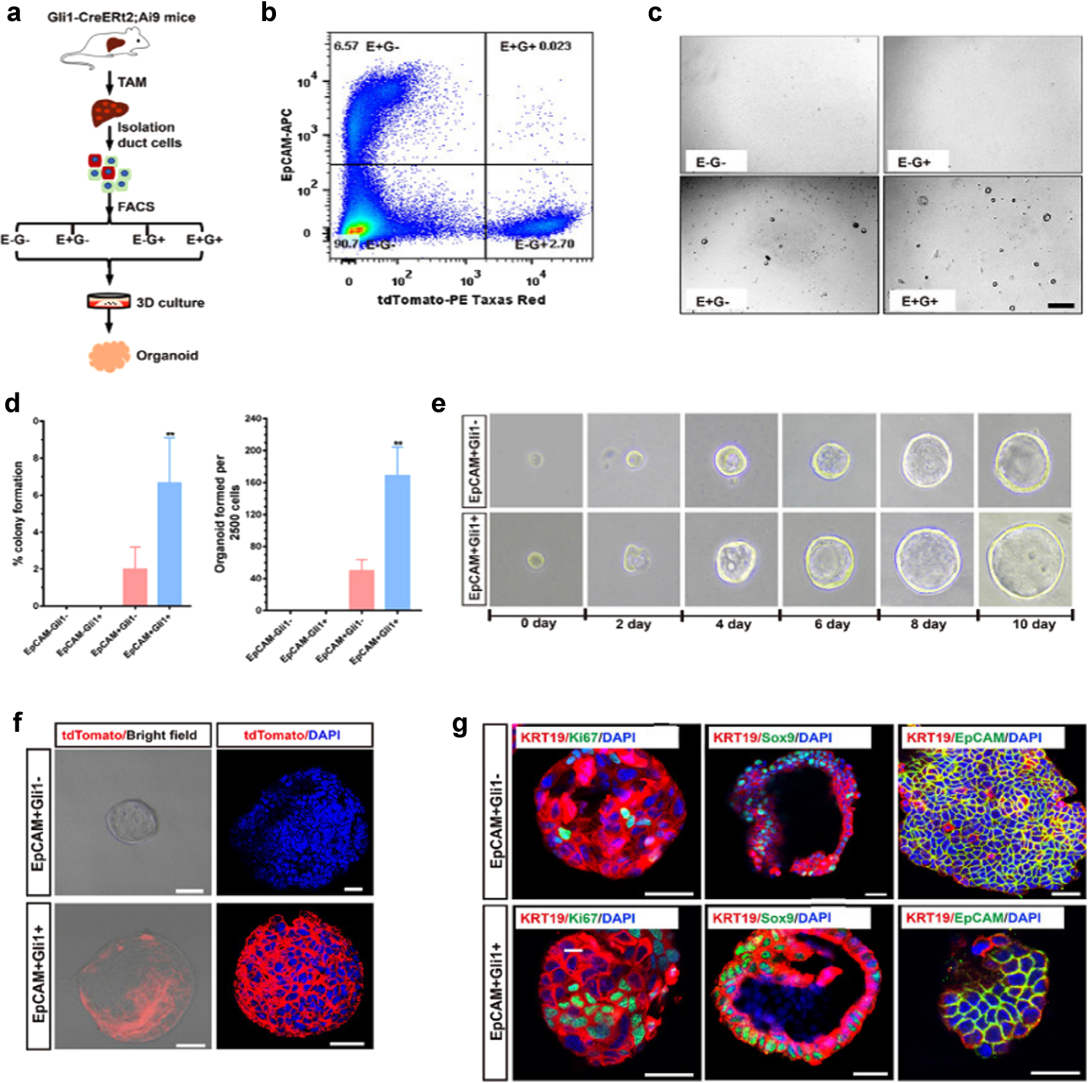
Liver organoid development from EpCAM^+^Gli1^−^ and EpCAM^+^Gli1^+^ single cells. **a** Schematic representation of organoid development from EpCAM^−^Gli1^−^, EpCAM^+^Gli1^−^, EpCAM^−^Gli1^+^ and EpCAM^+^Gli1^+^ single cells sorted by FACS. **b** FACS plot showing the expression of EpCAM and Gli1 in livers from Gli1-Cre^ERt2^;Ai9 mice after TAM induction. **c** Representative bright-field images of EpCAM^−^Gli1^−^, EpCAM^+^Gli1^−^, EpCAM^−^Gli1^+^ and EpCAM^+^Gli1^+^ cells cultured for 3 days as liver organoids. Original magnifications: 10×. **d** Percentage of the colony formation efficiency (left) and the numbers of organoids formed per 2500 single cells (right). Data are presented as means ± SEM (*n* = 3). **P < 0.01. **e** Representative images of initial single seeded EpCAM^+^Gli1^−^ and EpCAM^+^Gli1^+^ cells maintained in 3D culture for a period of 10 days. Original magnifications were 40× (days 0–4), 20× (days 6–8), and 10× (day 10). **f** Whole-mount organoid immunofluorescence staining for tdTomato (red) in organoids generated from EpCAM^+^Gli1^−^ and EpCAM^+^Gli1^+^ cells^.^ Scale bar, 50 μm. **g** Immunofluorescence staining for KRT19 (red) and Sox9 (green), EpCAM (green) or Ki67 (green) in organoids generated from EpCAM^+^Gli1^−^ and EpCAM^+^Gli1^+^ cells. Scale bar, 50 μm.

We next functionally analyzed EpCAM^+^Gli1^+^ cell population in liver organoid formation in vitro. We isolated EpCAM^+^Gli1^+^, EpCAM^+^Gli1^−^, EpCAM^−^Gli1^+^ and EpCAM^−^Gli1^−^ cells from Gli1-Cre^ERt2^;Ai9 mouse livers 2 weeks after TAM injection and assessed their organoid formation efficiency (Fig. 6a, b; Supplementary Fig. S7a). We found that EpCAM^+^Gli1^+^ cells exhibited a threefold increase in liver organoid forming efficiency compared with EpCAM^+^Gli1^−^ cells, whereas EpCAM^−^Gli1^−^ and EpCAM^−^Gli1^+^ cells did not form organoids (Fig. 6c, d). On day 10 of culture, the organoid-forming efficiency of EpCAM^+^Gli1^−^ and EpCAM^+^Gli1^+^ cells were 2% ± 0.98% and 6.67% ± 1.99%, respectively (Fig. 6d; Supplementary Fig. S7b). To confirm the cell source of cultured organoids, we performed immunofluorescent staining for tdTomato. We readily detected tdTomato fluorescence in whole-mount organoids cultured from EpCAM^+^Gli1^+^ cells (Fig. 6f). Organoids derived from EpCAM^+^Gli1^−^ and EpCAM^+^Gli1^+^ cells were morphologically similar to liver organoids that were previously derived from bile duct cells^34,35^ and allowed for serial passaging (Fig. 6e). Moreover, the organoid formation efficiency was not significantly different between the two groups after passage. Immunofluorescence staining revealed that EpCAM^+^Gli1^–^ and EpCAM^+^Gli1^+^ organoids expressed duct cell markers such as KRT19, and progenitor cell markers such as Sox9 (Fig. 6g). Moreover, immunohistochemical analysis of Ki67 in organoids showed many Ki67-positive cells (Fig. 6g), whereas the organoids failed to express mature hepatocyte markers such as Alb or HNF4α. To further validate these results, we sorted EpCAM^−^Gli1^−^, EpCAM^+^Gli1^−^, EpCAM^−^Gli1^+^, and EpCAM^+^Gli1^+^ cell populations from the liver of Gli1-LacZ mice and assessed organoid formation after 5 days of in vitro culture (Supplementary Fig. S7c, d). Consistently, we observed that only EpCAM^+^Gli1^−^ and EpCAM^+^Gli1^+^ cells could form organoids and EpCAM^+^Gli1^+^ cells exhibited the highest organoid forming efficiency (Supplementary Fig. S7e–g). These results demonstrated that the EpCAM^+^Gli1^+^ cell population is a new type of LPC that exhibits dual cell lineage characteristics in vitro.

**Fig. 7:**
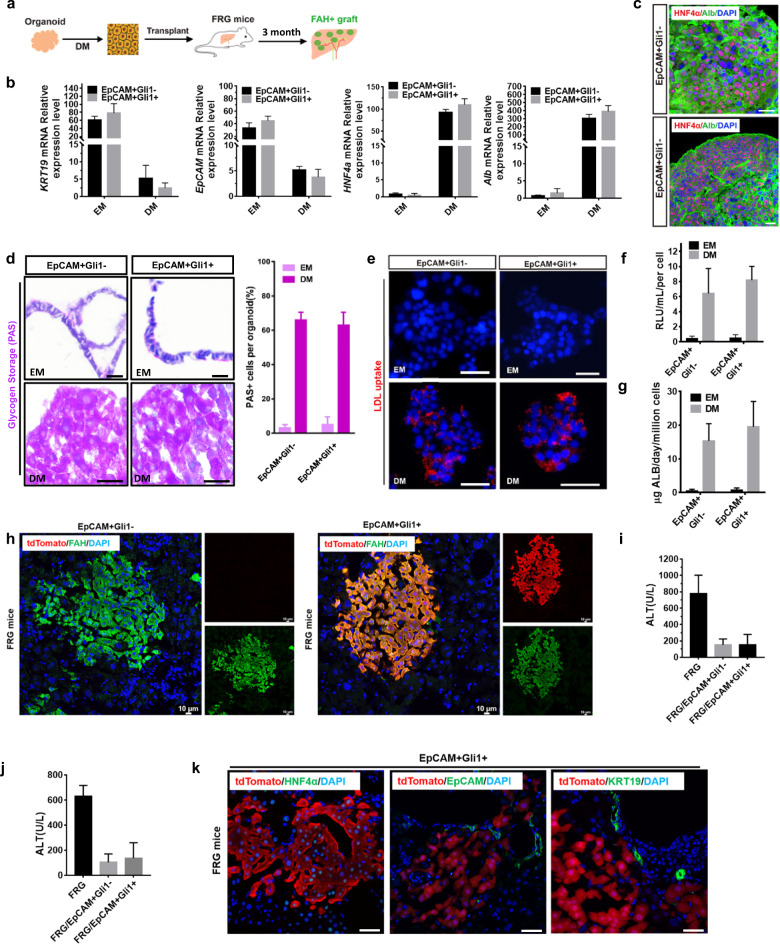
EpCAM^+^Gli1^+^ organoids differentiate into functional hepatocytes and efficiently repopulate FRG liver after transplantation. **a** Schematic illustration of the experimental design. **b** RT-qPCR analysis of *Krt19*, *EpCAM*, *Alb*, and *HNF4α* in expansion medium (EM) or differentiation medium (DM). Data are represented as means ± SEM (*n* = 3). **c** Immunofluorescence analysis of the hepatocyte marker genes *Alb* (green) or *HNF4α* (red) in DM. Scale bar, 50 μm. **d** Glycogen accumulation was determined by periodic acid-Schiff (PAS) staining in organoids grown in EM or DM for 12 days. Scale bar, 50 μm. **e** LDL uptake was analyzed using an LDL fluorescent substrate (red) in organoids that were maintained in EM or DM for 12 days. Scale bar, 50 μm. **f** Albumin secretion in EM or DM for 12 days. Data are shown as means ± SEM (*n* = 3). **g** Measurement of the cytochrome activity of CYP3A4 in EM or DM for 12 days. Relative light units (RLUs) per mL per million cells are shown. Data are presented as means ± SEM (*n* = 3). **h** Immunofluorescence staining for FAH (green) and tdTomato (red) showing the engraftment of FAH^+^ hepatocytes in FRG recipient livers at 3 months post transplantation. Scale bar, 10 μm. **i**, **j** Serum levels of ALT and AST were determined at 3 months post transplantation. Data are shown as means ± SEM (*n* = 3). **k** Immunofluorescence staining for tdTomato (red) and HNF4α (green), KRT19 (green), or EpCAM (green) at 3 months after transplantation. Scale bar, 50 μm.

To determine the ability of organoids to differentiate into mature hepatocytes, EpCAM^+^Gli1^−^ and EpCAM^+^Gli1^+^ cell-derived organoids were cultured in the differentiation medium (Fig. 7a). Organoids were positive for the hepatocyte markers Alb and HNF4α, but were negative for the cholangiocyte markers KRT19 and EpCAM (Fig. 7b, c). Furthermore, the organoids that were differentiated from EpCAM^+^Gli1^+^ cells had the ability to accumulate glycogen and uptake LDL in vitro (Fig. 7d, e). Liver functions of hepatocytes, such as albumin secretion and CYP3A4 activation, were significantly increased in the differentiation medium (Fig. 7f, g). These results demonstrated that EpCAM^+^Gli1^+^ cells have the potential to differentiate toward hepatocyte lineages.

### Hepatocytes differentiated from EpCAM^+^Gli1^+^ organoids efficiently repopulate FRG liver after transplantation

To evaluate whether differentiated organoid cells could be transplanted and repair injured liver tissues in vivo, we used FRG (Fah^−/−^Rag2^−/−^IL2rg^−/−^) mice^36^. The hepatocytes differentiated from EpCAM^+^Gli1^−^ or EpCAM^+^Gli1^+^ organoids were then digested by enzymes into single cells and transplanted into FRG mice by spleen injection. Subsequently, we analyzed engraftment by FAH staining at 3 months after transplantation. At 3 months, immunostaining for FAH and tdTomato showed significant engraftment (Fig. 7h). Serum levels of aspartate transaminase (ALT) and alanine transaminase (AST) were significantly reduced between the two groups after transplantation (no significance between EpCAM^+^Gli1^+^ and EpCAM^+^Gli1^−^), further confirming the improved liver functions in these mice (Fig. 7i, j). Next, we characterized whether EpCAM^+^Gli1^+^ cells underwent further maturation in vivo. Immunofluorescent staining showed that tdTomato^+^ maintained the expression of HNF4α and lost expression of KRT19 and EpCAM (Fig. 7k). These data suggested that hepatocytes differentiated from EpCAM^+^Gli1^+^ organoids efficiently repopulated the livers of FRG mice.

## Discussion

The liver is one of the largest and most important metabolic organs and exhibits a remarkably high potential to regenerate after injury^5,37^. Hepatocytes are a major functional cell type for liver physiological functions. Therefore, the cell source of new hepatocytes has been a main focus in research on liver regeneration after injury. Liver regenerative capacity is mainly based on the self-replication ability of the two main epithelial cell types: hepatocytes and cholangiocytes^38,39^. In acute and chronic injury models, these two cell populations cease to be quiescent and replicate to compensate for lost functional parenchyma. Lineage tracing studies demonstrated that hepatocytes contributed to biliary epithelial regeneration via transdifferentiation^11,12^. Similarly, biliary epithelial cells can also be converted into hepatocytes when hepatocytes are severely injured^3,13^. These data suggest that the plasticity of hepatocytes and cholangiocytes contributes to liver regeneration after injury. However, the role of nonepithelial cells, including mesenchymal cells and immune cells, in liver regeneration remains incompletely understood. Mesenchymal cells that surround ductular may not only influence the differentiation of progenitor cells, but also contribute to progenitor cell populations^40^. HSCs are liver-specific mesenchymal cells that play roles in liver physiology and fibrogenesis^41^. Previous studies on HSCs have mainly focused on the relationship with hepatic fibrosis; recently, it was suggested that HSCs are of great importance in hepatocyte proliferation, differentiation and maturation during liver regeneration^22,23,41,42^. Although in vitro culture experiments and transplantation provide evidence that HSCs are bipotent, their lineage potential needs further evaluation in vivo. In this study, we identified a new Gli1^+^ mesenchymal-like cell population that contributes to generation of new hepatocytes during chronic injury. We found that Gli1^+^ cells in the liver are a heterogeneous population that consists of PDGFRα^+^ stromal cells and a very small subset of EpCAM^+^ cells. scRNA-seq showed that EpCAM^+^Gli1^+^ cells appear to be in biphenotypic state, coexpressing epithelial markers and mesenchymal markers. This finding is consistent with a previous work showing that a small subset of Gli1^+^ cells are positive for the biliary epithelial cell marker EpCAM^43^. Notably, EpCAM^+^ cells are often identified as potential liver stem cells^5,31^, indicating that EpCAM^+^Gli1^+^ cells may be the source of Gli1^+^ hepatocytes after liver injury.

When hepatocytes and cholangiocytes are severely damaged, LPCs may contribute to liver regeneration by giving rise to new hepatocytes^5,9^. However, the existence, origin, fate, activation, and contribution of LPCs to regeneration in the liver are controversial due to the mixed results obtained from different lineage tracing studies^15,44–49^. There is no doubt that the identification of LPC-specific markers would greatly advance this field. Previous studies suggested that the mesenchymal marker Foxl1 could be a marker of facultative LPCs, which can produce hepatocytes and biliary cells after liver injury^20,21,50^. However, it should be emphasized that the *Foxl1* gene is not expressed prior to damage and thus cannot be used to prelabel the cells that arise during liver regeneration. Furthermore, the clonogenicity and differentiation potential of Foxl1^+^ cells have not yet been verified in vitro. scRNA-seq has recently proven that the EpCAM^+^TROP2^int^ population is a putative progenitor population in the adult human liver that is potentially involved in liver regeneration^8^. However, direct evidence showing that TROP2^+^ cells give rise to new hepatocytes during liver regeneration in vivo is still lacking. Our data suggest that Gli1 and EpCAM identify a specific cell population that exists in healthy liver, can be expanded as epithelial organoids in vitro and differentiate into functional hepatocytes both in vivo and in vitro. EpCAM^+^Gli1^+^ cells serve as a potential LPC and contribute to liver repair and regeneration. Therefore, Gli1 and EpCAM can be used as markers for screening LPCs.

The epithelial-mesenchymal transition (EMT) plays a crucial role in differentiation in multiple tissues and organs^51^. EMT also contributes to tissue repair but can adversely cause organ fibrosis. Primary EMT is followed by differentiation events that generate different cell types. The mesenchymal state is associated with the capacity of cells to migrate to sites of injury and maintain stemness, allowing their subsequent differentiation into multiple cell types during development and the initiation of metastasis^52^. In the liver, LPCs are capable of EMT during chronic liver injury, raising the possibility that EMT might be involved in liver regeneration^53^. Previous studies also showed that LPCs exhibit the features of the epithelial-mesenchymal lineage, by which the injured liver could be repaired^20,54^. We also found that EpCAM^+^Gli1^+^ cells exhibit a biphenotypic state, coexpressing epithelial and mesenchymal markers, associated with EMT. The expression of Gli1 in LPCs could be important for the acquisition of mesenchymal characteristics, such as enhanced motility and invasion, promoting the migration of progenitor cells to the site of liver injury.

Perivascular Gli1^+^ MSC-like cells could originate from myofibroblasts and play a central role in the fibrosis of solid organs (including the liver) after injury; ablating these cells could relieve fibrosis and rescue organ function^27^. Although the function of Gli1 is not indispensable in adult tissues, Gli1^+^ cells exhibit MSC properties and form a pericyte niche^55,56^. Genetic fate tracing experiments revealed that Gli1^+^ cells are a major source of organ myofibroblasts after injury^27^. Genetic ablation of Gli1^+^ cells improve fibrosis and rescues organ function^27,57^. In addition, Gli1^+^ cells have been regarded as stem cells that support calvarial bone turnover and injury in calvarial sutures^58^. Gli1^+^ cells, which are a component of Wnt-producing stem cell niche, also play an important role in the self-renewal of colonic epithelial stem cells^59^. In addition, other evidence suggests that Gli1^+^ capsular stem cells are capable of lineage conversion toward the steroidogenic lineage in the adrenal cortex at different ages^60^. In our study, we discovered a rare subpopulation of cells expressing Gli1, and these Gli1^+^ cells could produce hepatic cells after liver injury and participate in the process of liver regeneration. Overall, our results provide evidence for the heterogeneity of Gli1^+^ cells, which simultaneously exert adverse and advantageous effects after liver injury.

In the present study, we identified a novel rare progenitor cell population expressing both Gli1 and EpCAM, and these cells could participate in the liver repair process immediately after the occurrence of chronic hepatopathy. It is critical to explore the mechanism by which the identified cells regulate the liver repair process. Because Gli1-specific antibodies do not yet exist, exploring the cell surface proteins in the liver, which would be helpful for the easy identification of our newly discovered cells, will assist the research on human liver regeneration. Overall, the genetic evidence of EpCAM^+^Gli1^+^ cells in liver repair provides new insights into the cellular and molecular mechanisms of liver disease and regeneration.

## Materials and methods

### Mouse experiments

All animal studies were performed in accordance with the guidelines of the Institutional Animal Care and Use Committee of the Center for Excellence in Molecular Cell Science, Shanghai Institute of Biochemistry and Cell Biology, Chinese Academy of Sciences. Animals were housed in specific pathogen-free facilities at the Institute of Biochemistry and Cell Biology and kept under standard conditions with a 12 h day/night cycle and access to food and water ad libitum. Female and male (8–10 weeks old) mice of the following genotypes and strains were used: Gli1-LacZ (The Jackson Laboratory, stock# 008211, maintained on a C57BL/6 background), Gli1-Cre^ERt2^ (The Jackson Laboratory, stock# 007913, maintained on a C57BL/6 background), B6.Cg-*Gt(ROSA)26Sor*^*tm9(CAG-tdTomato)Hze*^/J (Ai9) (The Jackson Laboratory, stock# 007909, maintained on a C57BL/6 background), R26-YFP (The Jackson Laboratory, stock# 006148, maintained on a C57BL/6 background), HNF4α-Dre^ERt2^, R26-RSR-tdTomato and Ai66 reporter mice (maintained on 129×1/SvJ background)^14,61,62^, Tmprss2-Cre^ERt2^ (maintained on 129×1/SvJ background)^63^. FRG mice (obtained from Dr. Xin Wang and maintained on the mixed C57BL/6 J and 129S6/SvEvTac background)^36^. EpCAM-Cre^ERt2^ mouse line was generated using the CRISPR/Cas9 method by Shanghai Model Organisms Center, Inc. Briefly, a cDNA encoding Cre recombinase fused with a mutant form of the estrogen receptor hormone-binding domain (Cre^ERt2^) was targeted to the translational start codon ATG of *EpCAM* gene by homologous recombination. The Tmprss2-Dre^ERt2^ mice was generated using similar methodology and involved introduction of a 2A-Cre^ERt2^ cassette into the translational start codon ATG of the *Tmprss2* gene. For induction of all Cre- and Dre-recombinase models, TAM (Sigma) was diluted with corn oil (Sigma) to produce a TAM:corn oil mixture at 20 mg/mL. 8–10-week old mice were injected intraperitoneally with 200 μg/g body weight on three consecutive days to induce recombination. All lineage tracing experiments represent a minimum of *n* = 5 mice.

### Injury model

To induce liver injury, mice were fed with a diet supplemented with 0.1% (w/w) DDC for 4–12 weeks or were fed with CDE and MCD for 3 or 4 weeks. For CCl_4_-induced acute injury, mice were intraperitoneally injected with a single dose of 1 mL/kg body weight CCl_4_. For the CCl_4_-induced chronic injury model, CCl_4_ was dissolved at 1:3 in corn oil and injected intraperitoneally at a dose of 1 mL/g body weight every 3 days for ten times. Two-third PH was performed under 2% isoflurane anesthesia, and median laparotomy was performed followed by removal of the left lateral and the median lobe, as previously described^64^. Mice were euthanized 24 and 48 h after surgery.

### Immunofluorescence and β-gal staining

Freshly dissected mouse liver was fixed in 4% paraformaldehyde at 4 °C for 1 h. After fixation, tissues were washed in PBS for three times, dehydrated in 30% sucrose overnight at 4 °C and embedded in OCT. Cryosections (10 μm) were obtained and air-dried afterwards at room temperature. For staining, dried sections were washed in PBS and then blocked with 1% BSA and 0.05% Triton X-100 in PBS for 30 min at room temperature. Sections were incubated overnight at 4 °C with the primary antibodies listed in Supplementary Table S1. Primary antibodies were detected using fluorescent dye conjugated secondary antibodies (Alexa Fluor 488, Alexa Fluor 555 and Alexa Fluor 657; Invitrogen). Sections were stained with DAPI (4′,6-diamidino-2-phenylindole) and mounted with Aqua-Ploy/mount (Polysciences). Immunostaining images were acquired by Olympus fluorescence microscope (BX53) and Leica TCS SP8 confocal microscope. ImageJ software was used to analyze the collected images.

To detect β-gal activity, sections were rinsed in wash solution (0.1 M PBS containing 2 mM MgCl_2_, 0.02% NP-40, 0.01% Na-deoxycholate, pH 7.4) for three times and incubated in pre-warmed, filtered X-gal staining solution (wash solution supplemented with 5 mM K_3_Fe(CN)_6_, 5 mM K_4_Fe(CN)_6_, and 1 mg/mL X-gal) overnight in the dark at 37 °C. Prior to histological examination by light microscopy, some slides were counterstained with nuclear fast red staining solution following standard protocols.

### The CUBIC clearing

The CUBIC was performed as previously reported^65,66^. Briefly, the formaldehyde-fixed livers were resected into tissue blocks (3–5 mm × 3–5 mm), followed by wash with PBS. These liver blocks were immersed into 5 mL of 50% (v/v) CUBIC-L reagent (1:1 mixture of water:CUBIC-L) for 1 day and further immersed in 5 mL of CUBIC-L reagent for 5 days. Next, these livers were washed with PBS for 1 day and immersed in 5 mL of CUBIC-R+ reagent for 4 days. Liver fluorescence images were acquired with LiTone XL Light-sheet Microscope (LiT).

### Liver organoid

Liver from Gli1-cre^ERt2^;Ai9 and Gli1-LacZ mice was collected at 8–10 weeks of age and processed into a single-cell suspension using dispase II, collagenase IV and Dnase I, as previously described^67^. EpCAM^+^ and Gli1^+^ (tdTomato^+^ or CUG^+^) cells were identified via FACS sorting. For sorting and quantification, the following antibody was used: EpCAM-APC (eBioscience, Clone G8.8, 1:500). Isolated positive cells were mixed with Matrigel (BD Bioscience) and seeded and cultured as described previously^21^. Expansion medium was based on Advanced DMEM/F12 medium supplemented with 10 mM HEPES (Gibco), 2 nM GlutaMAX-1 (Gibco), 500× primocin (InvivoGen), 1× B27 (Gibco), 1.56 mM *N*-Acetylcysteine (Sigma), 10 mM Nicotinamide (Sigma), 0.5 μM A83-01 (Tocris), 10 μM Y27632 (Selleck), 50 ng/mL EGF (PeproTech), 10 ng/mL FGF10 (PeproTech), 1 ng/mL FGF2 (PeproTech), homemade R-Spondin (10%) and Noggin (10%) conditioned medium. To enhance hepatocyte cell fate, single cell-derived liver organoids were seeded and kept for 2–4 days in the liver expansion medium. Then the medium was changed to the differentiation medium: Advanced DMEM/F12 medium supplemented with 50 ng/mL EGF, 10 mM HEPES, 2 mM GlutaMAX-1, 500× primocin, 1× B27, 10 μM Y27632, 10 μM DAPT (Selleck), 25 ng/mL BMP7 (PeproTech), 25 ng/mL HGF (PeproTech) and 20 ng/mL Oncostatin M (R&D). For transplantation and in vitro functional studies, cultures were also supplemented with dexamethasone (30 mM) for the last 3 days of the differentiation. Medium was changed every other day for a period of 9–14 days.

### Functional analysis of organoid

To access glycogen storage, organoids in differentiation medium were embedded in paraffin. Sections (4 μm) were cut and stained using PAS staining kit (Solarbio) according to the manufacturer’s instructions. LDL uptake was detected with LDL Uptake Cell-Based Assay Kit (Cayman) according to the manufacturer’s instructions. Culture medium was harvested and albumin secretion was detected with mouse albumin ELISA kit (Bethyl). Organoids in differentiation medium were incubated with 3 mM of luciferin-IPA overnight for the measurement of CYP3A11 activity using the P450-Glo^TM^ CYP3A4 assay system (Promega).

### RNA isolation, reverse transcription and real-time PCR

Total RNA was extracted from organoids and liver using TRIZOl (Invitrogen) following the manufacturer’s instructions. RNA concentration and purity were determined by measuring the optical density (OD) at 260 nm and 280 nm wavelengths using NanoDrop. The OD_260/280_ ratios were > 1.8 and < 2.1 for all samples. The total RNAs (500 ng) from organoids and liver were used to convert mRNAs into cDNAs using ReverTra Ace qPCR RT master Mix with gDNA Remover kit (TOYOBO) according to the manufacturer’s instructions.

Real-time PCR was then performed in a 25 μL reaction volume that contains 12.5 μL of SYBR Green (Takara), 2 μL of template cDNA, and 1 μM of primers (Supplementary Table S2) using the ABI Fast 7500. The thermal cycling conditions were 95 °C for 10 min, followed by 40 cycles at 94 °C for 15 s, 60 °C for 30 s, and 72 °C for 30 s. *GAPDH* was employed for normalization. Each experiment was repeated independently at least three times, and the fold change in the expression of each gene was analyzed via a 2^−ΔΔCt^ method^68^.

### Transplantation assay

FRG mice were fed with drinking water containing 7.5 mg/L 2-(2-nitro-4-trifluoro-methyl-benzoyl)-1,3 cyclohexanedione (NTBC). A mix of 8–12-week old male and female mice were used for transplantation and no sex bias differences were detected. For transplantation, three clones derived from three different Gli1-cre^ERt2^;Ai9 mice were grown for at least 2 months. Cultures were kept in liver expansion medium and transferred to differentiation medium 9 days before transplantation. Suspensions of 1 × 10^6^ organoid-derived cells were injected intrasplenically to FRG mice. Mice were given the NTBC drug in drinking water for 4 days following transplantation. Then NTBC was removed and mouse health status and body weight were monitored every other day. The livers of transplanted mice were harvested at 3 months after transplantation and evaluated by FAH antibody staining. Serum AST and ALT were measured in livers of transplanted mice at 3 months after transplantation. Mice were sacrificed, blood was collected and serum was separated from the clotted blood by centrifugation at 12,000 rpm for 10 min. ALT and AST were measured using ALT and AST test kit from Abcam.

### scRNA-seq library preparation and sequencing

scRNA-seq library was constructed using Smart-seq2, as described previously^69^. Single cells were sorted in 96-well plates containing 2 µL of cell lysis buffer (0.2% Triton X-100 and RNase inhibitor), 1 µL of oligo-dT primer and 1 µL of dNTP mix. Next, we performed reverse transcription of polyadenylated transcripts using SuperScript II reverse transcriptase in the presence of a template switch oligonucleotide primer (TSO). The double-stranded RT-product was PCR amplified using Kapa Ready Mix (Kapa Biosystems) for 21 cycles, to yield the whole transcriptome amplification product. The amplification product was cleaned up with VAHTS DNA Clean Beads and QC with QIxcel (to confirm the correct product size) and Qubit (to determine quantity). Next, single-cell library was generated using TruePrep DNA Library Prep Kit V2 for Illumina from Vazyme. Each single-cell library was individually barcoded by PCR with index primers. The barcoded single cells were pooled and sequenced on an Illumina NextSeq sequencer.

### Processing of scRNA-seq data

Raw sequencing reads were firstly trimmed by trim_galore (TrimGalore-0.5.0) to remove adapters and TSO sequence. Clean reads were then mapped to mouse genome (Mus_musculus.GRCm38.84) by STAR (star-2.7)^70^. The featureCounts (subread-1.6.0-source)^71^ was performed to generate raw counts. Based on raw counts, the expression levels of each genes were normalized by log_2_(TPM/10 + 1) as described previously^72^.

### Further analysis of scRNA-seq

Downstream analysis and visualization were performed with R package Seurat (version 3.1.1)^73,74^. Only the genes expressed in > 10 cells were reserved, and the cells with > 1000 genes expressed were retained for downstream analysis. “FindVariableFeatures” was performed to detect variable genes across the single cells (selection.method = “vst”). The key parameters of “FindClusters” were set with resolution = 0.05. To further compare biological function among EpCAM^−^Gli1^+^ cells, EpCAM^+^Gli1^−^ cells, EpCAM^+^Gli1^+^ cells and hepatocytes, Metascape (http://metascape.org/gp/index.html)^75^ and GSEA^76^ were performed. Doublets were identified and filtered by DoubletDecon^77^ and DoubletFinder^78^.

### Statistical analysis

All data were presented as means ± SEM. The results were analyzed using one- or two-way analysis of variance, followed by a least significant difference tukey’s test, and *P* < 0.05 was considered statistically significant. All statistical analyses were performed in SPSS 17.0. The “*n*” in the study represented the number of biological replicates and was indicated.

## Supplementary information

Supplemental information
