# Supplementary material for: Identification of a rare Gli1+ progenitor cell population contributing to liver regeneration during chronic injury
Source: Cell Discov. 2022 Nov 1;8:118. doi: 10.1038/s41421-022-00474-3 (PMC9622734; doi:10.1038/s41421-022-00474-3)
Supplement: Supplementary file 1 — Supplemental information [file 41421_2022_474_MOESM1_ESM.pdf]

Supplemental Information

Supplementary Fig. S1 (Related to Fig. 1). Gli1$^+$ cell enlargement appears differently in different damage models.

a The distribution of Gli1$^+$ cells were evaluated in situ in X-gal-stained sections of livers from Gli1-LacZ mice. Scale bar, 50 μm. **b** Immunofluorescence co-staining for β-gal (green) with KRT19 and OPN (cholangiocyte marker, red), Alb and GS (hepatocyte marker, red), VE-CAD (endothelial marker, red) and F4/80 (macrophage marker, red) in liver sections. Slides were counterstained with DAPI (blue). Scale bar, 50 μm. **c** Immunostaining for β-gal (green), EpCAM (cholangiocyte marker, red), and α-SMA (activated HSC marker, red) in liver sections. Scale bar, 50 μm. Right panels: Quantification of the percentage of Gli1$^+$ cells expressing EpCAM and α-SMA. Means ± SEM (n = 3). **d** Experimental design and representative histological images of X-gal$^+$ cells in sections of sham or PH livers. Scale bar, 50 μm. Right panels: Quantification of the number of X-gal$^+$ cells in sham or PH livers. Means ± SEM (n = 5). **e** Representative histological images of X-gal$^+$ cells in liver sections and the number of X-gal$^+$ liver cells from control or CCl_4-treated mice. Scale bar, 50 μm. Right panels: Quantification of the number of X-gal$^+$ cells. Means ± SEM (n = 5). **f** Representative histological images of X-gal$^+$ cells in liver sections from control or 10× CCl_4-treated mice. Scale bar, 50 μm. Right panels: Quantification of the number of X-gal$^+$ cells. Means ± SEM (n = 5). $^*p < 0.05$. **g, h** Representative histological images of X-gal$^+$ cells in liver sections from mice fed a normal diet and a 3-week CDE (**g**) or MCD (**h**) diet. Scale bar, 50 μm. Right panels: Quantification of the number of X-gal$^+$ cells. Means ± SEM (n = 5). $^*p < 0.05$. $^{**}p < 0.01$. **i** Immunofluorescence staining for β-gal (green) in liver sections from mice fed a normal diet and a 4-week DDC, CDE or MCD diet. Scale bar, 50 μm.

Supplementary Fig. S2 (Related to Fig. 1). Cell labeling in Gli1-CreER; Ai9 mice with Oil. **a** Schematic showing the experimental design. **b** Immunostaining for tdTomato (red) in liver sections from Gli1-CreER; Ai9 mice treated with Oil at various time points. Scale bar, 200 μm.

Supplementary Fig. S3 (Related to Fig. 1). Gli1^{+} cells differentiate into hepatocytes during liver damage.

a Schematic illustration of the experimental design. **b-e** Immunostaining for tdTomato (red) in liver sections at various time points after TAM administration. Scale bar, 100 μm. **f** Quantification of the percentage of tdTomato^{+} cells in liver sections. Means ± SEM (n = 5). **g** Schematic showing the experimental design. **h, i** Immunostaining for tdTomato (red) in liver sections at various time points after DDC-induced injury. Scale bar, 100 μm. **j** Quantification of the percentage of tdTomato^{+} cells in liver sections. Means ± SEM (n = 5). *$p < 0.05$. **k** Immunostaining for tdTomato (red) and HNF4α (green) in serial liver sections after DDC-induced injury. Scale bar, 50 μm. **l** Immunostaining for tdTomato (red) and KRT19 (green) or EpCAM in 50 μm liver sections after 4 weeks of a normal diet or DDC diet. Scale bar, 50 μm. **m** Quantification of the percentage of tdTomato^{+} hepatocyte adjacent to the PV. Means ± SEM (n = 4)

Supplementary Fig. S4 (Related to Fig. 3). The cellular source of Gli1.

a RNAscope analysis of hepatic expression of *Gli1* (red) and EpCAM (green) co-staining on 8-week-old WT mice. PV, portal vein. Scale bar, 50 μm. **b** Immunofluorescence staining for tdTomato (red) and KRT19 (green) or Sox9 (green) in liver sections from Gli1-CreERt2; Ai9 mice after TAM administration. Scale bar, 50 μm. **c** Immunofluorescence staining for tdTomato (red) and F4/80 (green), HNF4α (green), Alb (green) or VE-CAD (green) in liver sections from Gli1-CreERt2; Ai9 mice after TAM administration. Scale bar, 50 μm. **d** Flow cytometric analysis of the percentage of tdTomato^{+} cells among EpCAM^{+} cells.

Supplementary Fig. S5 (Related to Fig. 4). Analysis of scRNA-seq data.

a Doublets were identified and filtered by DoubletDecon and DoubletFinder. **b** Principal component analysis (PCA) of the transcriptome data. **c-f** Violin plots showing the expression of select specific lineage-associated genes from the scRNA-seq data. **g** Signaling pathways that were significantly enriched in EpCAM^{+}Gli1^{+} cells using BioCarta gene sets for GSEA (p <0.05).

Supplementary Fig. S6 (Related to Fig. 5). Gli1$^+$ cells differentiate into hepatocytes during chronic injury.

a Experimental design for lineage tracing of EpCAM$^+$ cells using EpCAM-CreERt2; Ai9 mice. **b** Schematic showing the experimental strategy. **c** Immunostaining for tdTomato (red) and EpCAM (green) in intestinal sections. Scale bar, 50 µm. Right panels: Quantification of the percentage of tdTomato$^+$ cells expressing EpCAM. Means ± SEM (n = 3). **d** Immunostaining for tdTomato (red) and EpCAM (green) in intestinal sections and liver sections. PV, portal vein. Scale bar, 50 µm. Right panels: Quantification of the percentage of tdTomato$^+$ cells expressing EpCAM. Means ± SEM (n = 3). **e** Immunostaining for GFP (red) in liver sections from Tmprss2-CreERt2; R26-YFP mice. Scale bar, 50 µm. **f** Immunostaining for tdTomato (red) in liver sections from Tmprss2-DreERt2; R26-RSR-tdTomato mice treated with or without TAM. Scale bar, 50 µm. **g** Immunostaining for tdTomato (red) and EpCAM (Green) or HNF4α(Green) in tissue sections from livers after EpCAM-CreERt2; Ai9 mice received a DDC diet for 4 weeks. Scale bar, 50 µm. **h** Quantification of the percentage of tdTomato$^+$ cells expressing HNF4α. Means ± SEM (n = 5). **i** Immunostaining for PDGFRα (Green), tdTomato (red) and HNF4α (brilliant blue), in tissue sections from livers after PDGFRα-CreERt2; Ai9 mice received a DDC diet for 4 weeks. Scale bar, 50 µm. **j** Quantification of the percentage of tdTomato$^+$ cells expressing HNF4α. Means ± SEM (n = 5).

Supplementary Fig. S7 (Related to Fig. 6). Organoids cultured from single EpCAM$^+$Gli1$^+$ cells from Gli1-LacZ mice.

a FACS gating strategy to isolate EpCAM$^+$ and tdTomato$^+$ cells. **b** Table showing the numbers used to calculate the % colony formation. **c** Schematic representation of organoid-development from EpCAM$^-$Gli1$^-$, EpCAM$^+$Gli1$^-$, EpCAM$^-$Gli1$^+$ and EpCAM$^+$Gli1$^+$ single cells from Gli1-LacZ mice sorted by FACS. **d** FACS plot showing the expression of EpCAM and Gli1 in the liver in Gli1-LacZ mice stained with CUG and EpCAM. **e** Representative images showing 5,000 sorted cells that grew into liver organoids 5 days after sorting. **f** Table showing the numbers used to

calculate the % colony formation for Gli1-LacZ mice. **g** Numbers of organoids formed per 5,000 single cells (left) and percentage of colony formation efficiency (right). Means ± SEM (n = 3). $^{**}p < 0.01$.

Supplementary Fig. S1

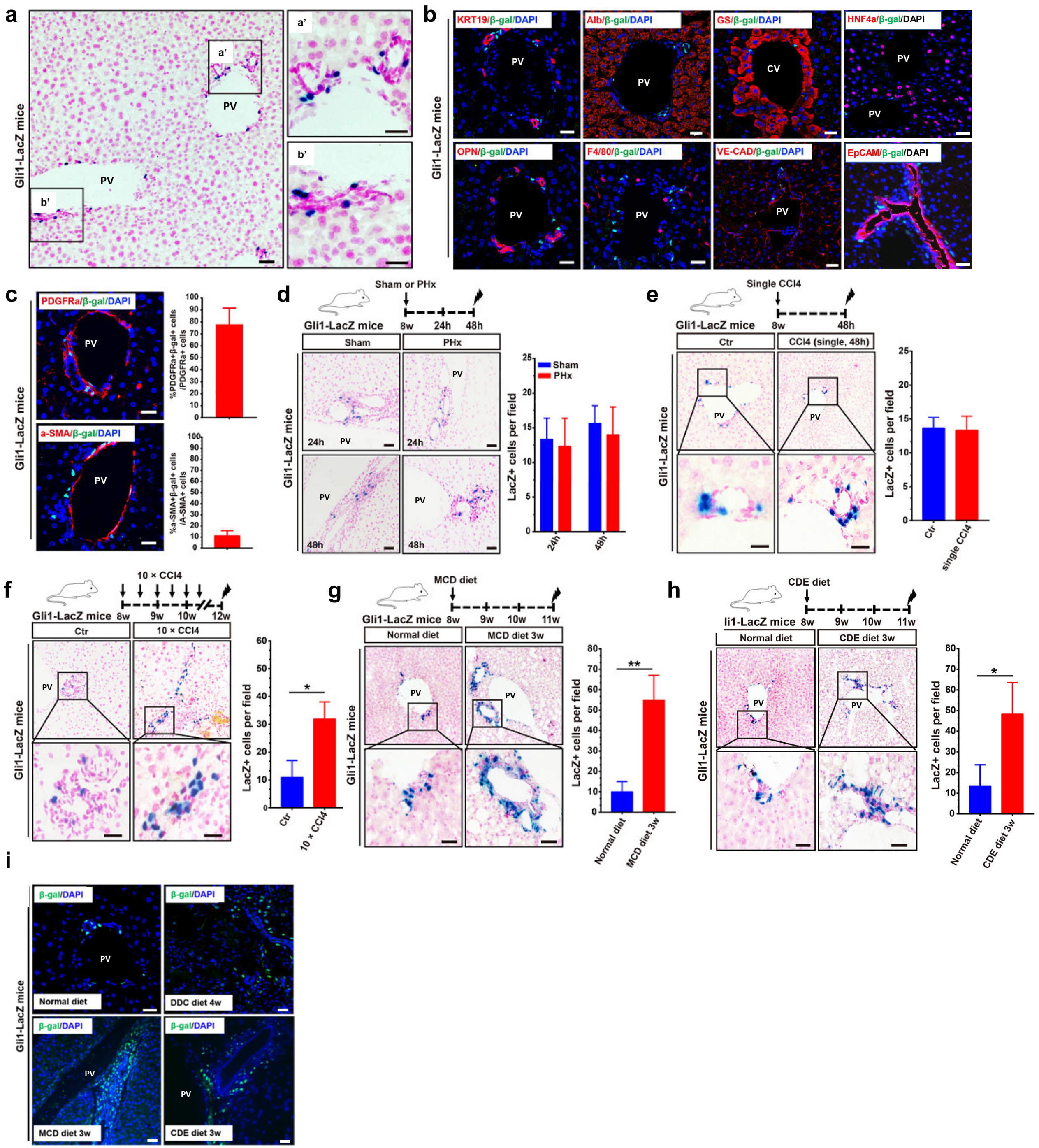

Supplementary Fig. S2

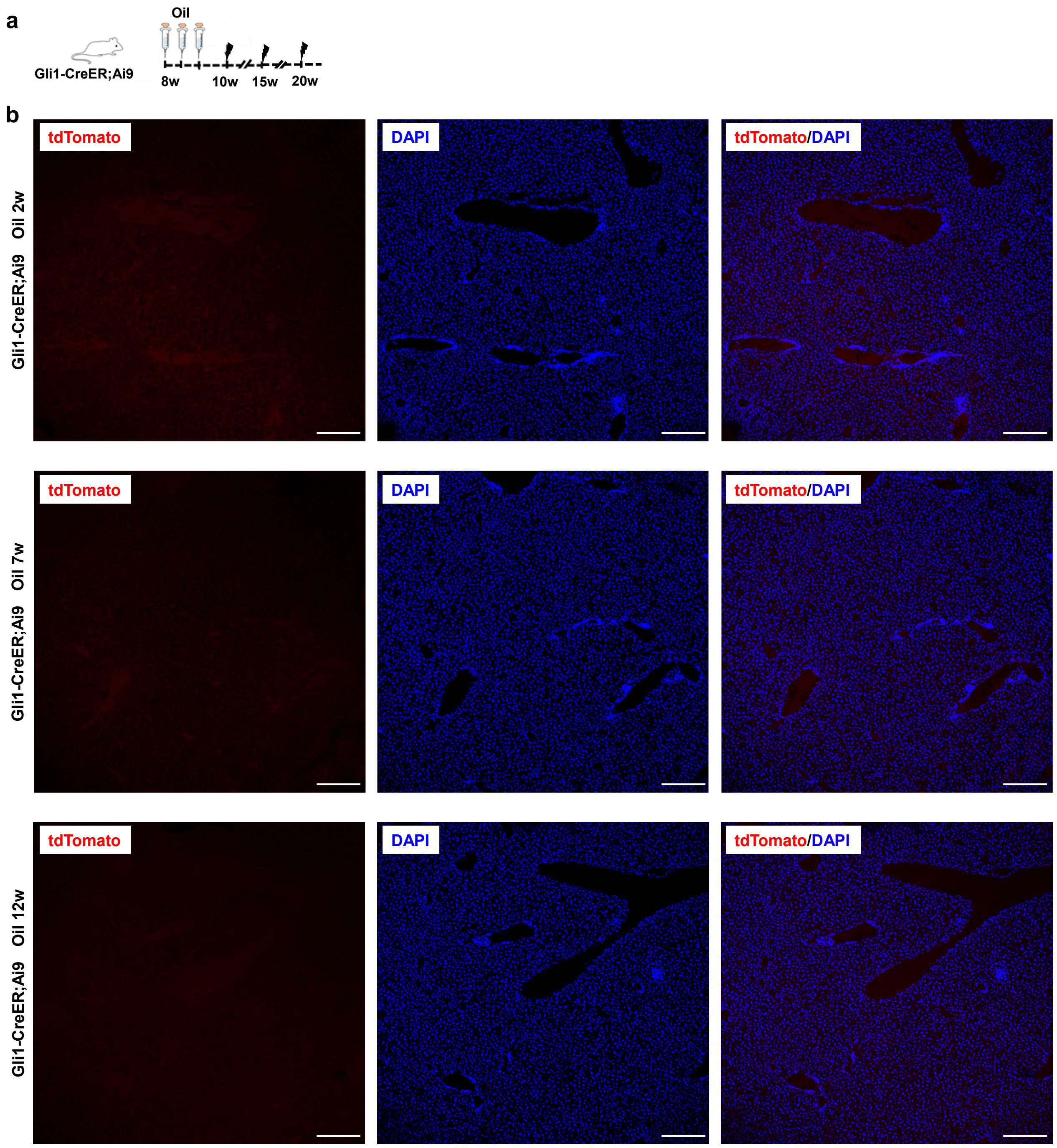

Supplementary Fig. S3

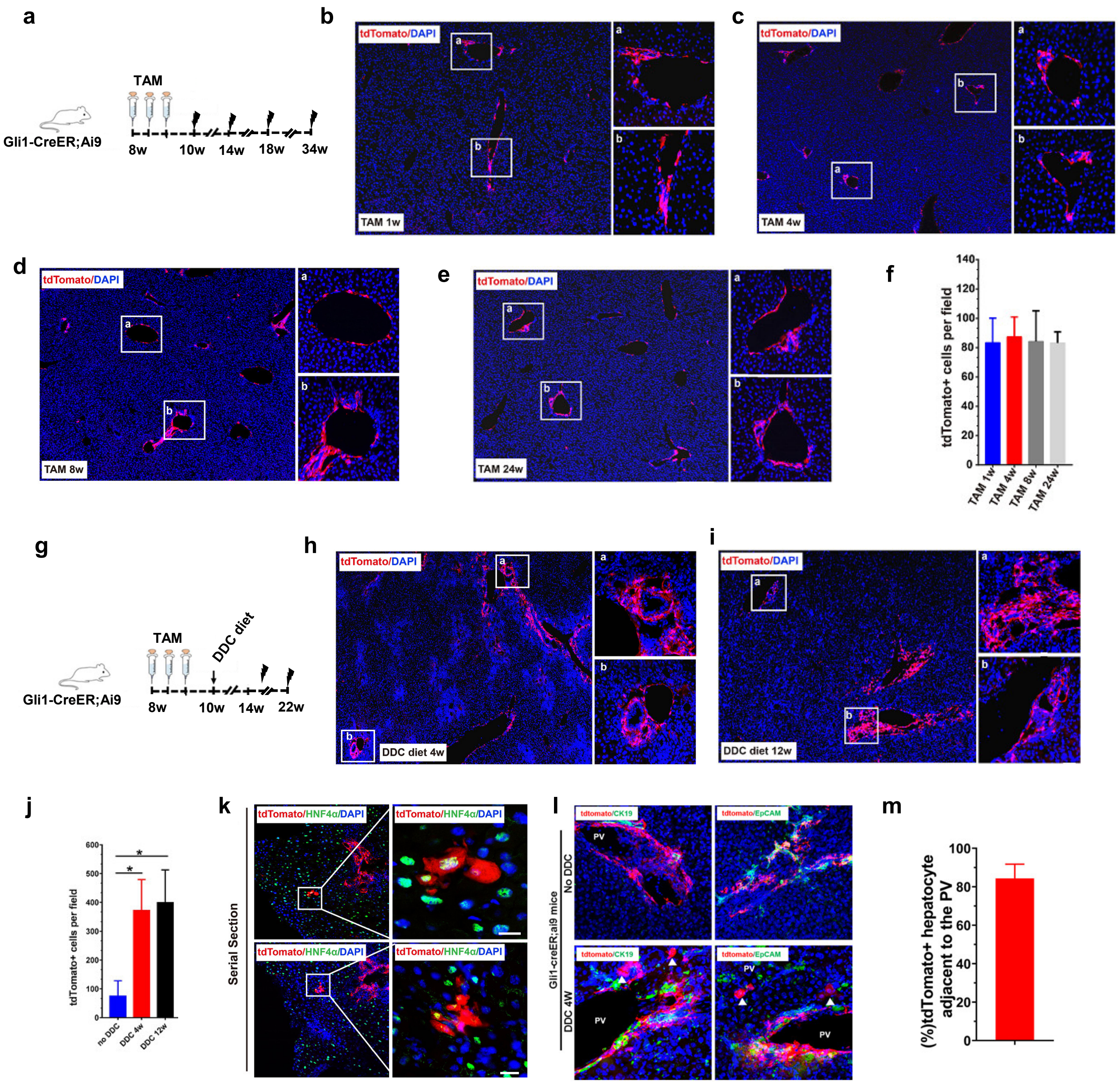

Supplementary Fig. S4

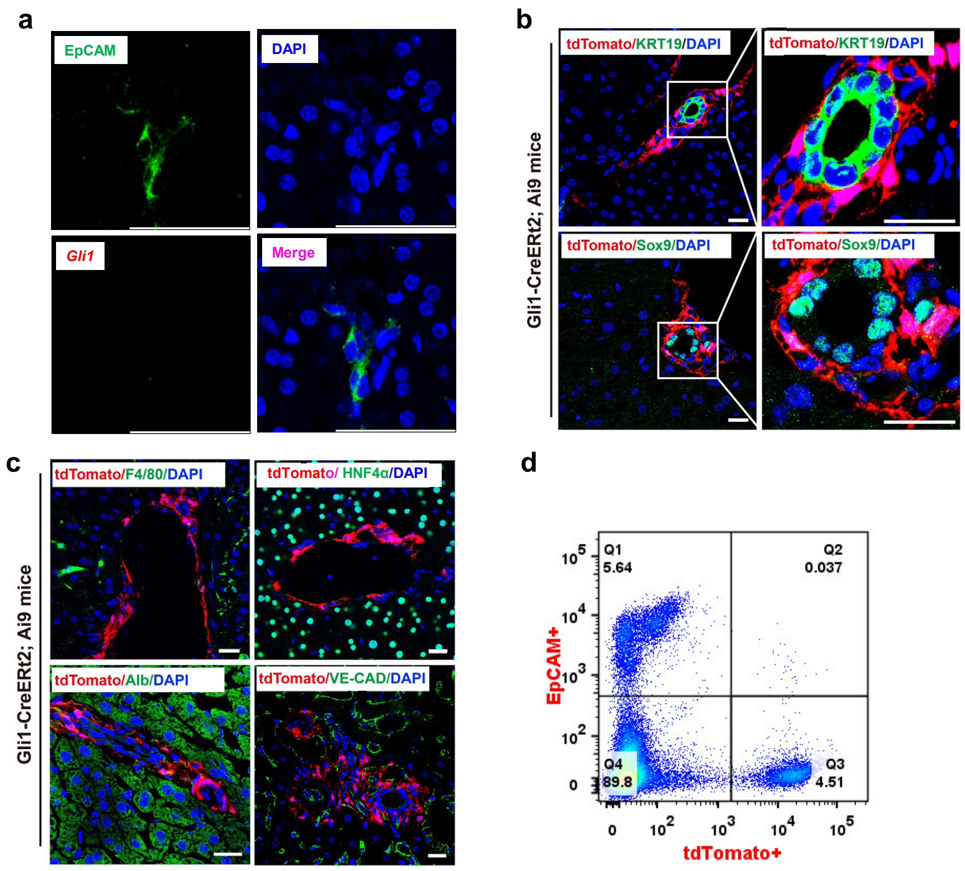

Supplementary Fig. S5

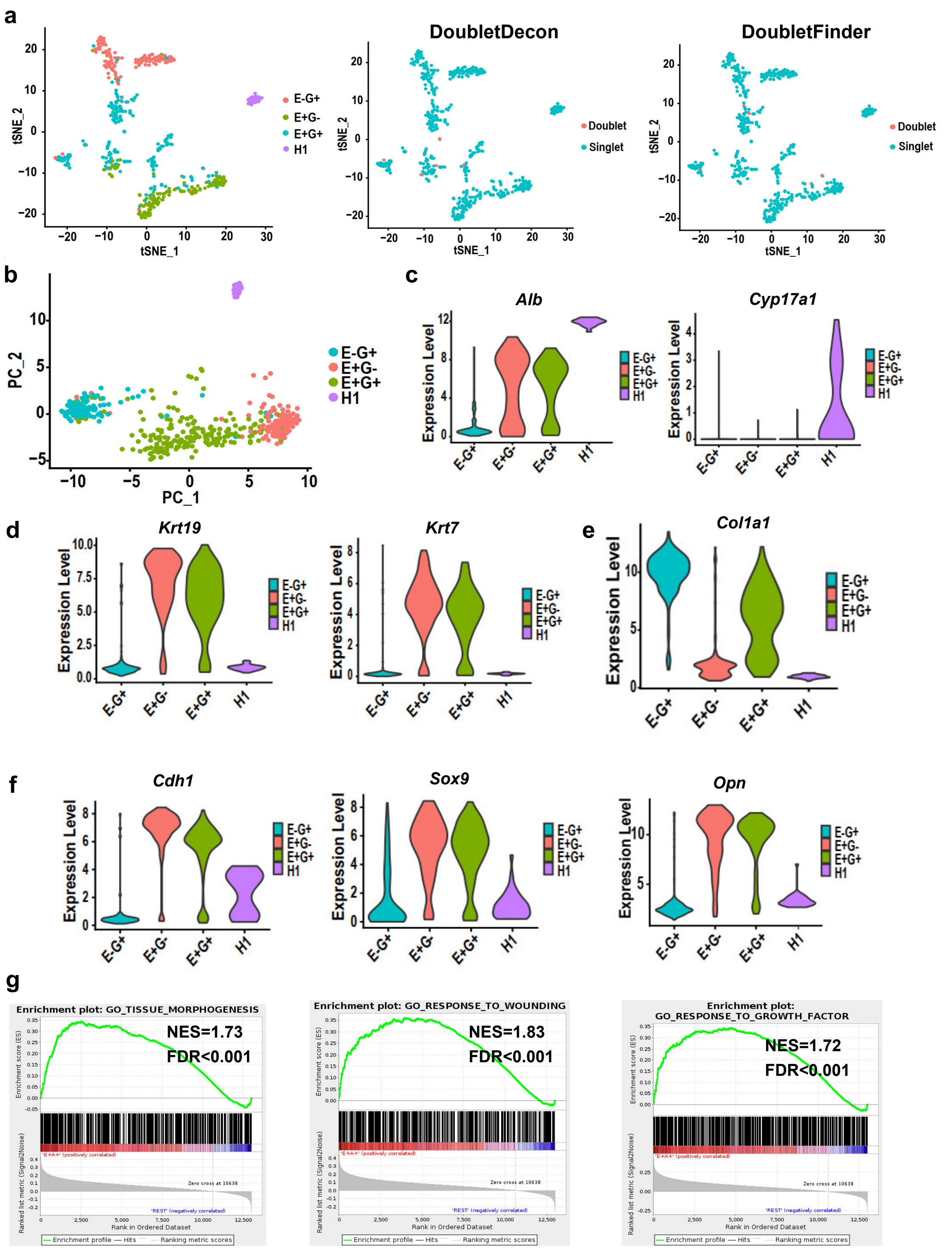

Supplementary Fig. S6

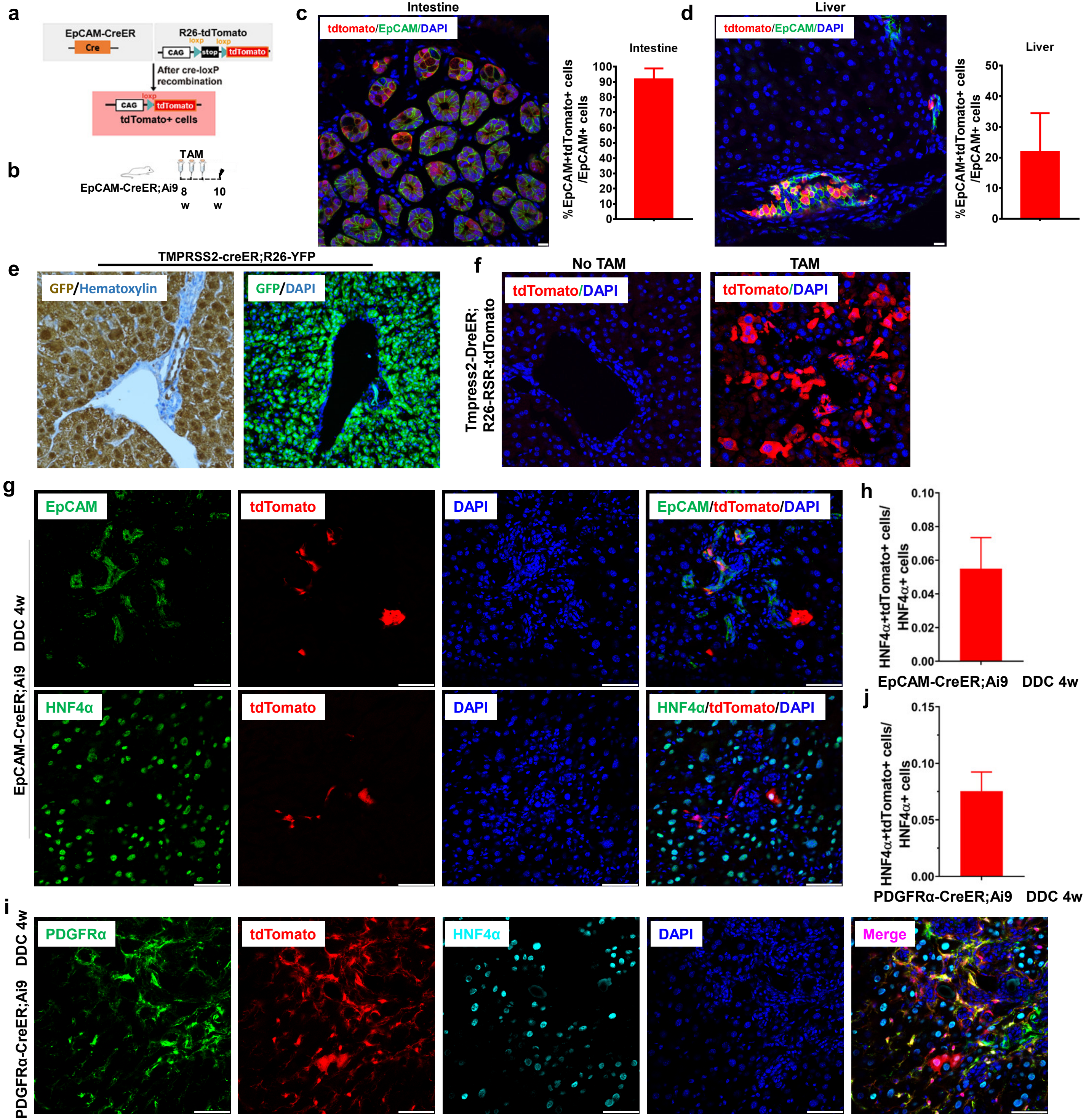

Supplementary Fig. S7

a

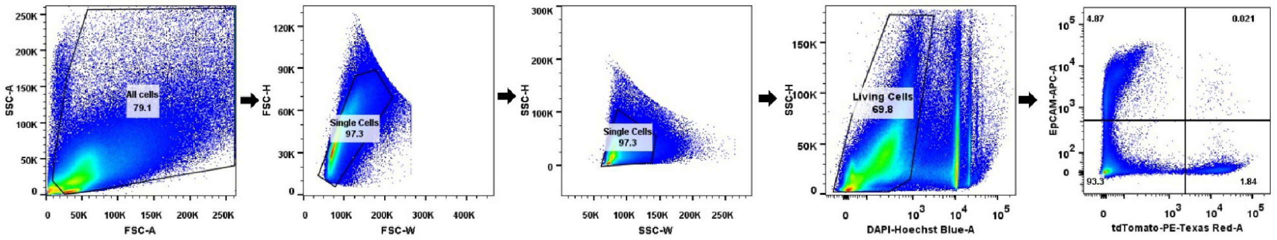

b

	Mouse1				Mouse2				Mouse3			
	E-G-	E-G+	E+G-	E+G+	E-G-	E-G+	E+G-	E+G+	E-G-	E-G+	E+G-	E+G+
n cells input (d0)	2500	2500	500	500	10000	5000	1000	1000	10000	1000	1000	1000
n organoids (d10)	0	0	10	44	0	0	32	72	0	0	8	40
% colony formation	0	0	2	8.8	0	0	3.2	7.2	0	0	0.8	4

c

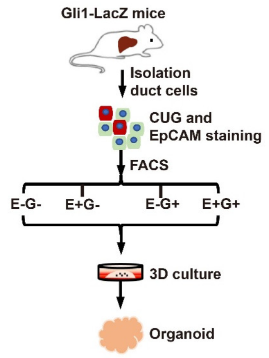

d

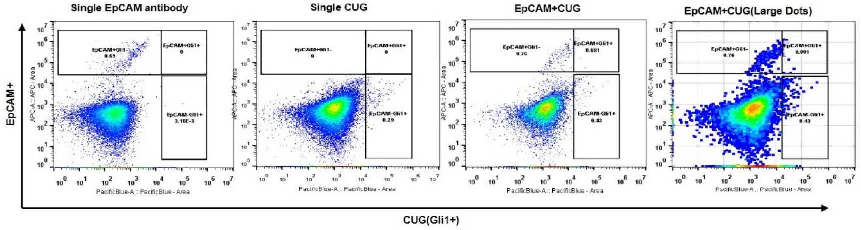

e

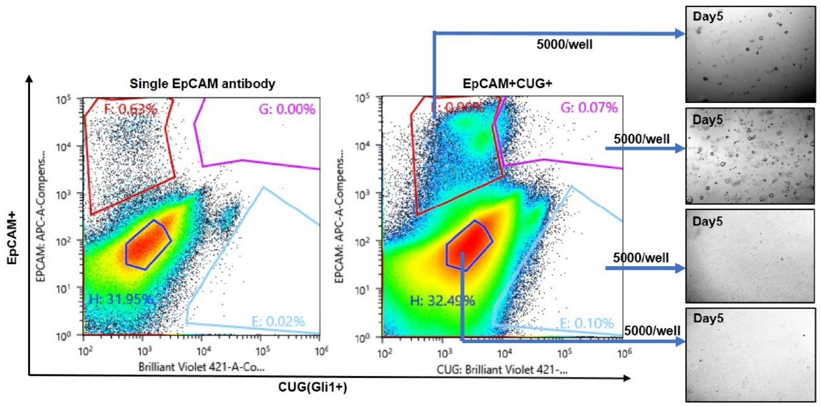

f

Name	Cell count / well	Organoid count
EpCAM-Gli1-	5000	0
EpCAM-Gli1+	5000	0
EpCAM+Gli1-	5000	28
EpCAM+Gli1+	5000	90

g

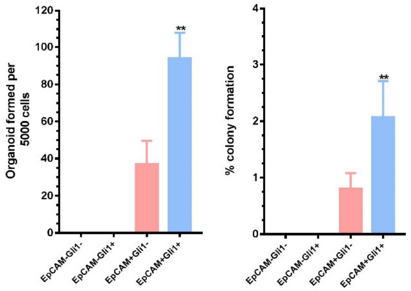

Supplementary Table 1: List of primary antibodies used for immunostaining experiments

Primary antibody	Species	Dilution	Source	Catalog number
HNF4α	rabbit	1/250	CST	3113S
E-cadherin	mouse	1/200	BD Biosciences	610182
CK19	rabbit	1/500	Abcam	ab52625
CK19 (Troma III)	rat	1/500	DSHB	AB_2133570
Osteopontin(OPN)	goat	1/100	R&D Systems	AF808
Sox9	rabbit	1/500	Millipore	AB5535
DsRed	rabbit	1/500	Clontech	632496
mcherry	goat	1/500	SICGEN	AB0081-200
PDGFRα	goat	1/100	R&D Systems	AF1062
VE-CAD	goat	1/100	R&D Systems	AF1002
EpCAM	rabbit	1/250	Abcam	ab71916
GS	mouse	1/1000	BD Biosciences	610517
Alb	mouse	1/250	Sigma	SAB3500217
β-gal	rabbit	1/250	Abcam	ab221199
α-SMA (Cy3-conjugated)	mouse	1/1000	Sigma	C6198
EpCAM (APC-conjugated)	rat	1/100	eBioscience	14-5791-82

Supplementary Table 2: List of primer sequences used for RT-qPCR

Gene	Pimer sequences (5'-3')
EpCAM	Fw - TGTGGACATAGCTGATGTGGCTTAC<br>Rv - CACCCTCAGGTCCATGCTCTTA
Krt19	Fw - GTCCTACAGATTGACAATGC<br>Rv - CACGCTCTGGATCTGTGACA
GAPDH	Fw - AGGTCGGTGTGAACGGATTTG<br>Rv - TGTAGACCATGTAGTTGAGGTCA
HNF4α	Fw - AGCTCGAGGCTCCGTAGTGTTT<br>Rv - GAAAATGTGCAGGTGTTGACCA
Alb	Fw - GCTGAGACCTTCACCTTCCA<br>Rv - TCTTCAGTTGCTCCGCTGTA
